# Supplementary material for: Who can I count on: Honor, self-reliance, and family in the United States and Iran
Source: PLoS One. 2024 Aug 27;19(8):e0306460. doi: 10.1371/journal.pone.0306460 (PMC11349206; doi:10.1371/journal.pone.0306460)
Supplement: S1 File — Supporting information, containing measures and exploratory and secondary analyses. (PDF) [file pone.0306460.s001.pdf]

Supplementary Materials

Data and analysis syntax for all studies are available on Open Science Framework (https://osf.io/m4sfn/?view_only=1f0d84a5426e4297b1292e3824b66941). This report contains the following sections: Study 1 Measures, Study 2a Measures, Study 2b Measures, Study 2b Measures, Descriptions of Measures for Secondary and Exploratory Analyses, Timeline of Studies, Factor Analyses, Other Preregistered Analyses, Analyses with Honor Subscales, Measurement Invariance, Regional Differences, Pre-Registered Mediation Analyses Controlling for Secondary Variables, Exploratory Analyses with Order of Scales.

Study 1 Measures

Likert-type items were rated on the following 7-point scale:

Strongly disagree, Disagree, Somewhat disagree, Neither agree nor disagree, Somewhat agree, Agree, Strongly agree

*Items with asterisks were removed from their scales for scoring and analyses.

Honor Values Scale (Novin & Oyserman, 2016):

Here are some opinions. Please read each and rate the extent to which you agree or disagree with each one.

1. I prefer to live with honor, even if it means I will earn less money.
2. To maintain my honor, I should not allow myself to be humiliated by others.
3. Even if I lost social status if I still have my honor, I can respect myself.
4. I would not disregard my honor even under tough life circumstances.
5. My honor will likely be negatively affected if I do not attend to my family obligations.
6. I would jeopardize the honor of my family if I behaved disgracefully.
7. It is my duty to defend the honor of my family.
8. It is important for me to keep face in front of others.
9. I would lose face if others saw me misbehave.
10. If I am embarrassed, I must not let it show or I will lose face.
11. My word is my bond; is how I feel; it would be dishonorable to behave otherwise.
12. Honorable people do not cheat people who trust them.
13. Loyalty is a core part of having honor.
14. Acting right is necessary to maintain my honor.

15. Reputation matters and should be vigorously defended.
16. My honor depends to a high degree on the appreciation and respect of others.
17. Disrespect damages honor.
18. I would show I had no honor if I didn't care what others would say.

Self-Reliance:

These are statements about not relying on official sources and institutions during the COVID-19 pandemic. For each, please rate how much you agree or disagree.

During this pandemic, instead of relying on official sources:

1. I should trust myself.
2. I should have confidence in my own intuitions.
3. I should take the actions I see fit.
4. I should follow my own intuition to protect myself and my family.

Independent Action/Adherence to Public Health Guidelines:

The COVID-19 pandemic suggests to some that the government can't be trusted to keep people safe during these uncertain times. Here are some actions people have been taking in response:

- Buying as much household cleaners and food as they can afford to stockpile
- Paying attention to medications to stockpile
- Taking on new sources of income for financial stability
- Staying home for as long as possible
- Purchasing guns/ammunition
- Purchasing medical-grade face masks (for example, N95's)
- Protesting
- Ignoring distancing requirements

Below, please rate the extent to which you agree with each of the statements regarding your own actions.

Because the government can't be trusted to keep people safe during the pandemic, I should:

1. stockpile household cleaners and food.
2. stockpile necessary medications.
3. take on new sources of income.*
4. stay home for as long as possible.
5. purchase guns or ammunition or check that my stock of guns and ammunition are adequate.
6. purchase medical-grade face masks.
7. attend protests.*
8. take other actions to protect myself and my family, with the assumption that the government won't be able to.

9. ignore distancing requirements.*

I should follow Centers for Disease Control (CDC) guidelines.

Strongly disagree, Disagree, Somewhat disagree, Neither agree nor disagree, Somewhat agree, Agree, Strongly agree

Government Distrust

These are statements about lack of trust in the government response to needs that arise during the pandemic. For each, please rate how much you agree or disagree.

During this pandemic, the government can’t be trusted to:

1. use federal powers to manufacture needed health and protective supplies.
2. protect the supply lines of food and other essential consumer products.
3. protect businesses from economic damage.
4. protect public safety.
5. confine its role and not overreach.

Confidence

Here are some statements about how much confidence you have in public institutions to respond effectively to the challenges of the pandemic. For each one, please rate how much you agree or disagree.

Regarding the challenges of COVID-19, I have confidence that:

the federal government is responding effectively.

my state government is responding effectively.

my local government is responding effectively.

the Centers for Disease Control (CDC) is responding effectively.

the healthcare system is responding effectively.

Here are some statements about how much you trust public institutions to provide you with accurate information in a timely manner about effects and consequences of COVID-19. For each one, please rate how much you agree or disagree.

Regarding COVID-19, I can trust:

the federal government to provide accurate information in a timely manner.

my state government to provide accurate information in a timely manner.

my local government to provide accurate information in a timely manner.

the Centers for Disease Control (CDC) to provide accurate information in a timely manner.

the healthcare system to provide accurate information in a timely manner.

General Knowledge

Earlier we told you about the concern people have that the government can't be trusted to keep them safe during the pandemic.

Prior to this survey, have you heard or read about the concern that the government can't be trusted to keep people safe during the pandemic?

Yes

No

Here are some actions people have taken in response to the concern that the government can't be trusted to keep them safe during the pandemic. Prior to this survey, which of these actions have you heard or read about? You may select more than one option.

Stockpiling household cleaners and food

Paying attention to medications to stockpile

Taking on new sources of income

Staying home for as long as possible

Purchasing guns/ammunition

Purchasing medical-grade face masks (for example, N95's)

Attending protests

Here are some guidelines that the Centers for Disease Control (CDC) has issued during the pandemic. Prior to this survey, which of these guidelines have you heard or read about? You may select more than one option.

Wearing cloth face coverings when outside the home

Staying home as much as possible

Washing hands frequently

Keeping a distance of at least 6 feet from others when outside the home

Avoid touching your face when outside the home

Cleaning frequently used surfaces such as doorknobs and cell phones

Attention Check

Please select the right-most option.

Strongly disagree, Disagree, Somewhat disagree, Neither agree nor disagree, Somewhat agree, Agree, Strongly agree

Political Orientation and Engagement

Would you say you lean liberal or conservative on social issues? In terms of social issues, I am...

Very liberal, Quite liberal, Slightly liberal, Moderate, Slightly conservative, Quite conservative, Very conservative (7 point scale)

Would you say you lean liberal or conservative on economic issues? In terms of economic issues, I am...

Very liberal, Quite liberal, Slightly liberal, Moderate, Slightly conservative, Quite conservative, Very conservative (7 point scale)

Did you vote in the 2016 United States presidential election?

Yes

No

(if yes is selected) Who did you vote for in the 2016 United States presidential election?

Donald Trump

Hillary Clinton

Gary Johnson

Jill Stein

Darrell Castle

Evan McMullin

Other (Please specify)

(if no is selected) Please check the reason you decided not to vote in the 2016 United States presidential election.

- I was not registered
- I was not eligible to vote
- I did not find a candidate I wanted to vote for
- Other (Please specify)

Demographics

What is your gender?

- Male
- Female
- Other

What is your age?

What state or territory do you live in?

What is your 5-digit ZIP code?

What race-ethnicities do you consider yourself? You may select more than one option.

- Asian/Asian American
- Caucasian/White
- Black/African American
- Hispanic/Latino
- Middle-Eastern
- Native American or Alaska Native
- Hawaiian Native or other Pacific Islander
- Other (please specify)

In your own words, please give your own description of your ethnic or racial background.

Study 2a Measures

Items from key, secondary, and exploratory measures were rated on the following 7-point scale:

Strongly disagree, Disagree, Somewhat disagree, Neither agree nor disagree, Somewhat agree, Agree, Strongly agree

Items in red were removed from their scales for scoring and analyses.

Honor Values Scale (Novin & Oyserman, 2016)

Here are some opinions. Please read each and rate the extent to which you agree or disagree with each one.

1. I prefer to live with honor, even if it means I will earn less money.
2. To maintain my honor, I should not allow myself to be humiliated by others.
3. Even if I lost social status if I still have my honor, I can respect myself.
4. I would not disregard my honor even under tough life circumstances.
5. My honor will likely be negatively affected if I do not attend to my family obligations.
6. I would jeopardize the honor of my family if I behaved disgracefully.
7. It is my duty to defend the honor of my family.
8. It is important for me to keep face in front of others.
9. I would lose face if others saw me misbehave.
10. If I am embarrassed, I must not let it show or I will lose face.
11. My word is my bond; is how I feel; it would be dishonorable to behave otherwise.
12. Honorable people do not cheat people who trust them.
13. Loyalty is a core part of having honor.
14. Acting right is necessary to maintain my honor.
15. Reputation matters and should be vigorously defended.
16. My honor depends to a high degree on the appreciation and respect of others.
17. Disrespect damages honor.
18. I would show I had no honor if I didn't care what others would say.

Self-Reliance

These are statements about not relying on official sources and institutions during the COVID-19 pandemic. For each, please rate how much you agree or disagree.

During this pandemic, instead of relying on official sources:

1. I should trust myself.

2. I should have confidence in my own intuitions.
3. I should take the actions I see fit.
4. I should follow my own intuition to protect myself and my family.

Independent Action

The COVID-19 pandemic suggests to some that the government can't be trusted to keep people safe during these uncertain times. Please rate the extent to which you agree or disagree with each of the statements.

I should:

1. decide for myself if I can trust the government to keep people safe during these uncertain times.
2. stockpile what I think is necessary, no matter what the government says.
3. seek out protections that make sense to me, no matter what the government says.
4. seek out information from sources other than the government.
5. decide for myself how serious COVID is.
6. take other actions to protect myself and my family, with the assumption that the government won't be able to.

Adherence to Public Health Guidelines

During the pandemic, I should:

1. follow Centers for Disease Control (CDC) guidelines.
2. stay home as much as possible.
3. try to convince my loved ones to stay home as much as possible.

Government Distrust

These are statements about lack of trust in the government response to needs that arise during the pandemic. For each, please rate how much you agree or disagree.

During the pandemic, the government can’t be trusted to:

1. use federal powers to manufacture needed health and protective supplies.
2. protect the supply lines of food and other essential consumer products.
3. protect businesses from economic damage.
4. protect public safety.
5. confine its role and not overreach.
6. restrain itself from infringing on the rights of individuals.*

Political Orientation and Engagement

How would you describe your political orientation?

I am a Republican

I am an Independent

I am a Democrat

(if Independent is selected) Would you say that you:

Lean Republican

Lean Democratic

Would you say you lean liberal or conservative on social issues? In terms of social issues, I am...

Very liberal, Quite liberal, Slightly liberal, Moderate, Slightly conservative, Quite conservative, Very conservative (7 point scale)

Would you say you lean liberal or conservative on economic issues? In terms of economic issues, I am...

Very liberal, Quite liberal, Slightly liberal, Moderate, Slightly conservative, Quite conservative, Very conservative (7 point scale)

Did you vote in the 2016 United States presidential election?

Yes

No

(if yes is selected) Who did you vote for in the 2016 United States presidential election?

Donald Trump

Hillary Clinton

Gary Johnson

Jill Stein

Darrell Castle

Evan McMullin

Other (Please specify)

(if no is selected) Please check the reason you decided not to vote in the 2016 United States presidential election.

I was not registered

I was not eligible to vote

I did not find a candidate I wanted to vote for

Other (Please specify)

Do you plan to vote in the 2020 Presidential election?

Yes

No (not eligible, too young, not a citizen)

No (could vote, but not registered)

No (could vote, but don't like my choices)

(if yes is selected) Which party do you plan to vote for in the 2020 election?

Democratic

Republican

Libertarian

Green

Other party or candidate (Please specify)

I don’t know

Attention Check

Please select the right-most option.

Strongly disagree, Disagree, Somewhat disagree, Neither agree nor disagree, Somewhat agree, Agree, Strongly agree

Demographics

What is your gender?

Male

Female

Other

What is your age?

What state or territory do you live in?

What is your 5-digit ZIP code?

What race-ethnicities do you consider yourself? You may select more than one option.

Asian/Asian American

Caucasian/White

Black/African American

Hispanic/Latino

Middle-Eastern

Native American or Alaska Native

Hawaiian Native or other Pacific Islander

Other (please specify)

In your own words, please give your own description of your ethnic or racial background:

Study 2b Measures

Items from key, secondary, and exploratory measures were rated on the following 7-point scale:

Strongly disagree, Disagree, Somewhat disagree, Neither agree nor disagree, Somewhat agree, Agree, Strongly agree

We provide both the Farsi items and their English translations.

Self-Reliance

These are statements about not relying on official sources and institutions during the COVID-19 pandemic. For each, please rate how much you agree or disagree.

جملات زیر در مورد اتکا به مجاری رسمی و دولتی در زمان کرونا است. لطفا میزان توافق یا عدم توافق خود را با هر جمله مشخص کنید.

During this pandemic, instead of relying on official sources:

در زمان کرونا، به جای تبعیت از مجاری رسمی:

1. I should trust myself.

   باید به خودم اعتماد داشته باشم.

2. I should have confidence in my own intuitions.

   باید به نظرات و حدسیات خودم مطمین باشم.

3. I should take the actions I see fit.

   هر کاری که خودم صلاح میدانم را انجام میدهم.

4. I should follow my own intuition to protect myself and my family.

   باید از نظرات خودم پیروی کنم تا بتوانم از خودم و خانواده ام محافظت کنم.

Independent Action

The COVID-19 pandemic suggests to some that the government can't be trusted to keep people safe during these uncertain times. Please rate the extent to which you agree or disagree with each of the statements.

به نظر برخی افراد کرونا نشان داد که به بعضی دولت ها نمی توان اطمینان کرد تا از مردم محافظت کنند. میزان توافق یا عدم توافق خود با جمله های زیر را مشخص کنید.

I should:

1. decide for myself if I can trust the government to keep people safe during these uncertain times.

   باید خودم شخصا تصمیم بگیرم که آیا به دولت اطمینان بکنم یا خیر.

2. stockpile what I think is necessary, no matter what the government says.

   باید مواد ضروری را ذخیره کنم، بدون توجه به چیزهایی که دولت می گوید.

3. seek out protections that make sense to me, no matter what the government says.

   باید ابزار محافظتی که نیاز میدانم را تهیه کنم، بدون توجه به چیزهایی که دولت می گوید.

4. seek out information from sources other than the government.

   باید دنبال اطلاعاتی بگردم که منبع آنها دولت نیست.

5. decide for myself how serious COVID is.

باید خودم تصمیم بگیرم که خطر کرونا چقدر جدی است.

6. take other actions to protect myself and my family, with the assumption that the government won't be able to.

باید خودم کارهایی انجام دهم تا از خودم و خانواده ام محافظت کنم چون دولت نمی تواند از ما محافظت کند.

Adherence to Public Health Guidelines

During the pandemic, I should:

1. follow the Ministry of Health and Medical Education guidelines.

در طول کرونا، باید از توصیه های وزارت بهداشت پیروی کنم.

2. stay home as much as possible.

در طول کرونا باید تا جایی که امکان دارد در خانه بمانم.

3. try to convince my loved ones to stay home as much as possible.

در طول کرونا، باید تلاش کنم که خانواده ام را راضی کنم که خانه بمانند.

Government Distrust

These are statements about lack of trust in the government response to needs that arise during the pandemic. For each, please rate how much you agree or disagree.

در این قسمت جملاتی راجع به اعتماد به دولت در مورد رفع نیازهای مردم آمده است. میزان توافق یا عدم توافق خود با جمله های زیر را مشخص کنید.

During the pandemic, the government can’t be trusted to:

در طول کرونا، نمی توان به دولت اطمینان کرد که:

1. use federal powers to manufacture needed health and protective supplies

در طول کرونا، نمی توان به دولت اطمینان کرد که: از قدرت دولتی خود استفاده کند و تجهیزات مورد نیاز نظام سلامت را تولید کند.

2. protect the supply lines of food and other essential consumer products.

در طول کرونا، نمی توان به دولت اطمینان کرد که: از خط تولید مواد خوراکی و باقی مایحتاج محافظت کند.

3. protect businesses from economic damage.

در طول کرونا، نمی توان به دولت اطمینان کرد که: از مشاغل نوپا و اقتصاد کشور محافظت کند.

4. protect public safety.

در طول کرونا، نمی توان به دولت اطمینان کرد که: از امنیت مردم محافظت کند.

5. confine its role and not overreach.

در طول کرونا، نمی توان به دولت اطمینان کرد که: بیش از اندازه در زندگی مردم دخالت نکند.

Honor Values Scale (Novin & Oyserman, 2016; Farsi translation by Atari, Graham, & Dehghani, 2020)

Here are some opinions. Please read each and rate the extent to which you agree or disagree with each one.

1. I prefer to live with honor, even if it means I will earn less money.

ترجیح میدهم با شرافت زندگی کنم، حتی اگر به معنای درآمد کمتر باشد

2. To maintain my honor, I should not allow myself to be humiliated by others.

به منظور حفظ آبرویم، نباید اجازه دهم که دیگران مرا تحقیر کنند.

3. Even if I lost social status if I still have my honor, I can respect myself.

حتی اگر جایگاه اجتماعی ام را از دست بدهم ولی آبرویم حفظ شود، میتوانم به خودم احترام بگذارم.

4. I would not disregard my honor even under tough life circumstances.

از شرافت خود در شرایط سخت زندگی هم دست برنخواهم داشت.

5. My honor will likely be negatively affected if I do not attend to my family obligations.

اگر به تعهدات خانوادگی خود پایبند نباشم، احتمالا شرف و آبرویم لکه دار خواهد شد.

6. I would jeopardize the honor of my family if I behaved disgracefully.

اگر رفتار نامناسبی داشته باشم، آبروی خانوادگی ام به خطر میافتد.

7. It is my duty to defend the honor of my family.

وظیفه من دفاع از آبروی خانوادهام است.

8. It is important for me to keep face in front of others.

مهم است که در مواجهه با دیگران وجهه خود را حفظ کنم.

9. I would lose face if others saw me misbehave.

اگر دیگران مرا با رفتار نامناسبی ببینند، وجهه خود را از دست میدهم.

10. If I am embarrassed, I must not let it show or I will lose face.

اگر شرمنده باشم، نباید آن را نشان دهم، چون باعث میشود وجه هام خدشه دار شود.

11. My word is my bond; is how I feel; it would be dishonorable to behave otherwise.

من معتقدم «مرد است و قولش» و اگر کسی برخلاف قولش عمل کند کار نادرستی کرده است.

12. Honorable people do not cheat people who trust them.

آدمهای با شرافت، به کسی که به آنها اعتماد کرده، خیانت نمیکنند.

13. Loyalty is a core part of having honor.

وفاداری بخش اساسی شرافت است.

14. Acting right is necessary to maintain my honor.

انجام دادن کار درست برای حفظ شرافتم لازم است.

15. Reputation matters and should be vigorously defended.

آبرو مهم است و باید شدیدا از آن دفاع کرد.

16. My honor depends to a high degree on the appreciation and respect of others.

شرافت من تا حد زیادی بستگی به تقدیر و احترام دیگران به من دارد.

17. Disrespect damages honor.

حفظ نکردن احترامها، به شرافت فرد صدمه میزند.

18. I would show I had no honor if I didn't care what others would say.

اینکه «مردم چی میگن...» برای من مهم است.

Political Orientation

How would you describe your political orientation?

نظرات سیاسی خود را به کدام نزدیک تر میدانید؟

Left-leaning

نزدیکتر به اصلاح طلب

Right-leaning

نزدیکتر به اصولگرا

Demographics

What is your sex?

جنسیت:

What is your age?

سن:

Study 3 Measures

Items from key, secondary, and exploratory measures were rated on the following 7-point scale:

Strongly disagree, Disagree, Somewhat disagree, Neither agree nor disagree, Somewhat agree, Agree, Strongly agree

Honor Values Scale (Novin & Oyserman, 2016; Farsi translation by Atari, Graham, & Dehghani, 2020)

Here are some opinions. Please read each and rate the extent to which you agree or disagree with each one.

1. I prefer to live with honor, even if it means I will earn less money.

   ترجیح میدهم با شرافت زندگی کنم، حتی اگر به معنای درآمد کمتر باشد

2. To maintain my honor, I should not allow myself to be humiliated by others.

   به منظور حفظ آبرویم، نباید اجازه دهم که دیگران مرا تحقیر کنند.

3. Even if I lost social status if I still have my honor, I can respect myself.

   حتی اگر جایگاه اجتماعی ام را از دست بدهم ولی آبرویم حفظ شود، میتوانم به خودم احترام بگذارم.

4. I would not disregard my honor even under tough life circumstances.

   از شرافت خود در شرایط سخت زندگی هم دست برنخواهم داشت.

5. My honor will likely be negatively affected if I do not attend to my family obligations.

   اگر به تعهدات خانوادگی خود پایبند نباشم، احتمالا شرف و آبرویم لکه دار خواهد شد.

6. I would jeopardize the honor of my family if I behaved disgracefully.

   اگر رفتار نامناسبی داشته باشم، آبروی خانوادگی ام به خطر میافتد.

7. It is my duty to defend the honor of my family.

   وظیفه من دفاع از آبروی خانوادهام است.

8. It is important for me to keep face in front of others.

   مهم است که در مواجهه با دیگران وجهه خود را حفظ کنم.

9. I would lose face if others saw me misbehave.

   اگر دیگران مرا با رفتار نامناسبی ببینند، وجهه خود را از دست میدهم.

10. If I am embarrassed, I must not let it show or I will lose face.

اگر شرمنده باشم، نباید آن را نشان دهم، چون باعث میشود وجه هام خدشه دار شود.

11. My word is my bond; is how I feel; it would be dishonorable to behave otherwise.

من معتقدم «مرد است و قولش» و اگر کسی برخلاف قولش عمل کند کار نادرستی کرده است.

12. Honorable people do not cheat people who trust them.

آدمهای با شرافت، به کسی که به آنها اعتماد کرده، خیانت نمیکنند.

13. Loyalty is a core part of having honor.

وفاداری بخش اساسی شرافت است.

14. Acting right is necessary to maintain my honor.

انجام دادن کار درست برای حفظ شرافتم لازم است.

15. Reputation matters and should be vigorously defended.

آبرو مهم است و باید شدیدا از آن دفاع کرد.

16. My honor depends to a high degree on the appreciation and respect of others.

شرافت من تا حد زیادی بستگی به تقدیر و احترام دیگران به من دارد.

17. Disrespect damages honor.

حفظ نکردن احترامها، به شرافت فرد صدمه میزند.

18. I would show I had no honor if I didn't care what others would say.

اینکه «مردم چی میگن...» برای من مهم است.

Self-Control Moralization

People differ in what they find morally relevant. Below is a list of actions. As you read each action, ask yourself, to what extent do I disagree or agree that this item is morally relevant. Your responses can range from **strongly disagreeing** to s**trongly agreeing** that the action is **morally relevant**. Focus on yourself, not what you think others might say. Tell us whether the action is relevant to morality at all -- not whether it is moral or immoral.

1. Giving in to distractions.
2. Feeling too tired to do laundry, so lying around in dirty clothes.
3. Being inactive when I have work to do.
4. Packing for a trip at the last minute.
5. Taking an elevator rather than walking up a single flight of stairs.
6. Being lazy when I have something to do.
7. Procrastinating

8. Doing my work at the last minute.
9. Letting responsibilities pile up.
10. Sitting around when I have work to do.
11. Choosing to wake up late, despite having a busy day ahead.
12. Wasting time in general.
13. Buying a car without doing research on price or quality.
14. Relying on others to fulfill my obligations for me.
15. Spending despite knowing I cannot afford it without help from others.
16. Asking to be taught instead of learning on my own.

Autonomy Subscale

People differ in how they feel in their everyday lives at work and school and in their relationships. Please read each of the following statements and rate how much you agree or disagree with each as it pertains to your own experiences in your own life, at work or school and in your relationships.

In my own life …

1. I can make a lot of inputs to deciding how things gets done
2. I feel pressured,
3. I am free to express my ideas and opinions.
4. I have to do what I am told.
5. People take my feelings into account.
6. I can pretty much be myself.
7. There is not much opportunity for me to decide for myself how to do what I need to do.

Family Reliance Scale

Here are some views about family relationships. Please read each and rate the extent to which you agree or disagree with each one.

1. There is nothing wrong with asking your family for support when you are in need.
2. Relatives should help each other, so it's acceptable to rely on them for help.
3. Even when they disagree, relatives should stand together and help one another.
4. Information is more trustworthy when they come from family members.
5. Protecting family from harm is one of the most important responsibilities in life.
6. One should do everything they can to ensure that their family has the resources to live well.

Demographics (Iran)

What is your sex?

جنسیت:

What is your age?

سن:

Religiosity

Education

Demographics (U.S.)

What is your gender?

Male

Female

Other

What is your age?

What state or territory do you live in?

What race-ethnicities do you consider yourself? You may select more than one option.

Asian/Asian American

Caucasian/White

Black/African American

Hispanic/Latino

Middle-Eastern

Native American or Alaska Native

Hawaiian Native or other Pacific Islander

Other (please specify)

Descriptions of Measures for Secondary and Exploratory Analyses

Study 1

COVID-19 Independent Action

We created 7 items describing actions that ignore government recommendations (e.g., "stockpile household cleaners and food"). Preliminary analyses revealed that they scaled into a 5-item independent action scale.

Government Distrust during COVID-19

We created a 5-item scale (e.g., "During this pandemic, the government can't be trusted to protect businesses from economic damage").

Confidence

For exploratory purposes, we created 10 items measuring confidence that 5 entities (federal government, state government, local government, CDC, and the healthcare system) can respond to COVID-19 effectively and provide accurate information about COVID-19 promptly.

Political Orientation and Engagement

Participants reported their political orientation in social and economic domains, and 2016 presidential election voting behavior.

Demographics

Gender, age, race-ethnicity, state of residence, and ZIP code.

General Knowledge and Attention Check

We asked participants about their prior knowledge of COVID-19 guidelines and actions people have taken in response to COVID-19. We included an attention check among these items, in which we asked participants to select the right-most option on a scale of 1 (*Strongly Disagree*) to 7 (*Strongly Agree*). Participant awareness of COVID-19 guidelines is shown in Table 1 below.

Table 1

Awareness of CDC-Issued COVID-19 Guidelines (Study 1)

Guideline	% Sample Aware
Wearing cloth face coverings when outside the home	94
Staying home as much as possible	96
Washing hands frequently	98
Keeping a distance of at least 6 feet from others when outside the home	95
Avoid touching your face when outside the home	96
Cleaning frequently used surfaces such as doorknobs and cell phones	85

Order of Scales

We randomized the order of the COVID-19 behavior scale, self-reliance/government distrust scales, and confidence scale. We randomly assigned order of presentation of the honor scale, with half of our participants completing the Honor Values Scale (HVS) items in 3 blocks – one block of 6 items before the distrust and self-reliance scales, one before the COVID-19 behavior scale, and one before the confidence scale. The other half completed all 18 items after completing the other scales. Political, demographic, and attention check items appeared at the end of the survey.

Study 2a

We omitted Confidence and General Knowledge items. The demographic items and government distrust scale remained constant from Study 1.

Independent Action

Given the rapid changes in the pandemic and anticipating that we would conduct a study in Iran, we created 6 items reflecting general-response patterns (e.g., "I should seek out protections that make sense to me, no matter what the government says.").

Political Orientation and Engagement

In addition to Study 1 items, we asked about political party affiliation and projected 2020 presidential election voting choices.

Attention Check

We included the attention check among the Political Orientation and Engagement items.

Order of Scales

We randomly assigned the order of the HVS: half of our participants completed it before the other questionnaires, and the other half completed it after. The other questionnaires appeared in this order: Self-Reliance, Independent Action, Adherence to Public Health Guidelines, and Government Distrust. Attention Check, Political Orientation and Engagement, and Demographics appeared last.

Study 2b

We omitted the Confidence items, General Knowledge items, and items about political engagement, race-ethnicity, and location. Self-Reliance, Independent Action, Government Distrust, and the Honor Values Scale items did not change from items administered to the Study 2a sample and were translated into Farsi.

Political Orientation

We omitted political party items and changed the two items on political orientation in economic and social domains to a single item measuring binary orientation (*left-leaning*, *right-leaning*).

Order of Scales

Items appeared in this order: Self-Reliance, Independent Action, Adherence to Public Health Guidelines, Government Distrust, Honor Values Scale, Political Orientation, and Demographics.

Timeline of Studies

Figures 1 and 2 indicate the timeline of Studies 1 through 2b on a graph indicating number of COVID cases over time (based on available COVID-19 cases data; Dong et al., 2020; Ritchie et al., 2020). Additionally, Iranian data for Study 3 was collected in July of 2022, while U.S. data for Study 3 was collected January of 2024.

Figure 1

Timeline of Data Collection and New COVID-19 Cases in the U.S., 2020-2021 (Studies 1 and 2a)

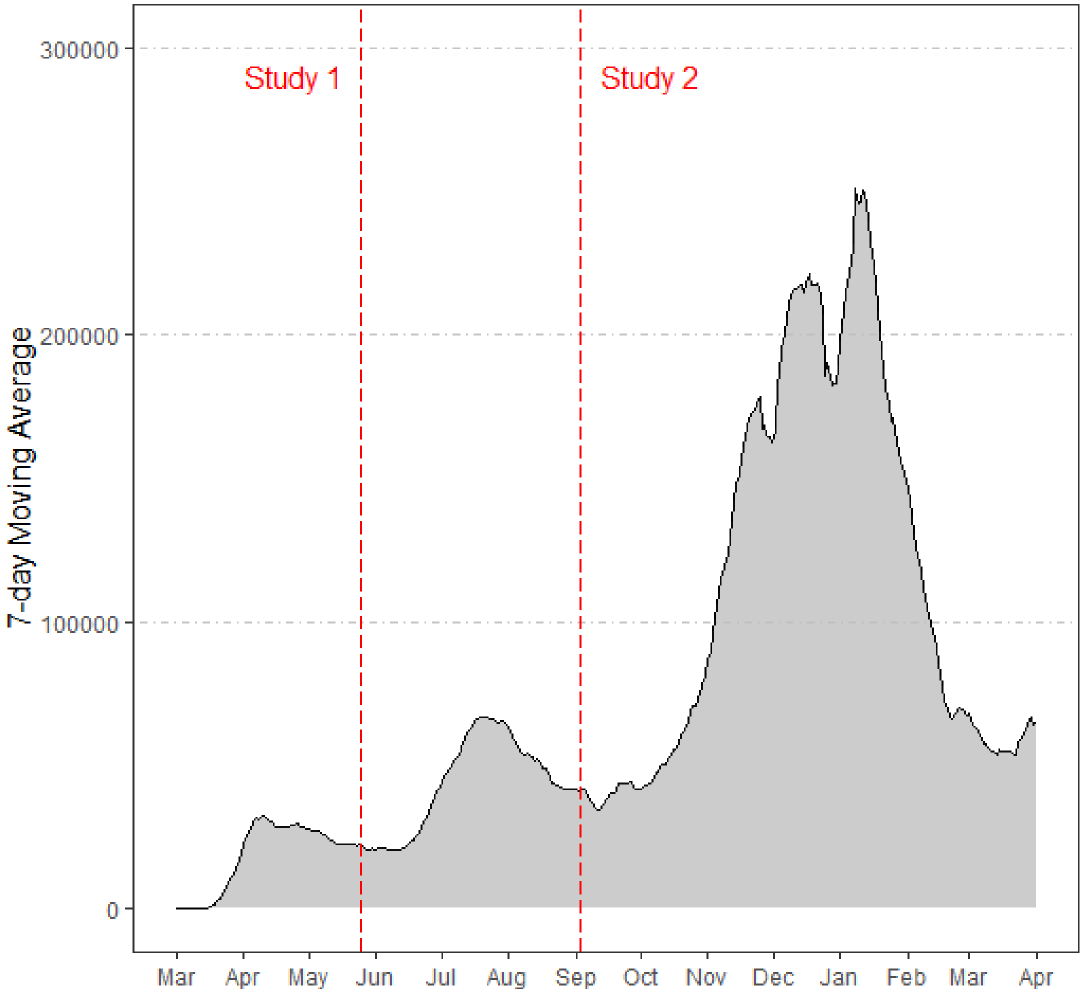

Note. The y-axis represents the 7-day moving average of new COVID-19 cases in the U.S. The

red lines indicate when data collection for Studies 1 and 2a began.

Figure 2

Timeline of Data Collection and New COVID-19 Cases in Iran, 2020-2021 (Study 2b)

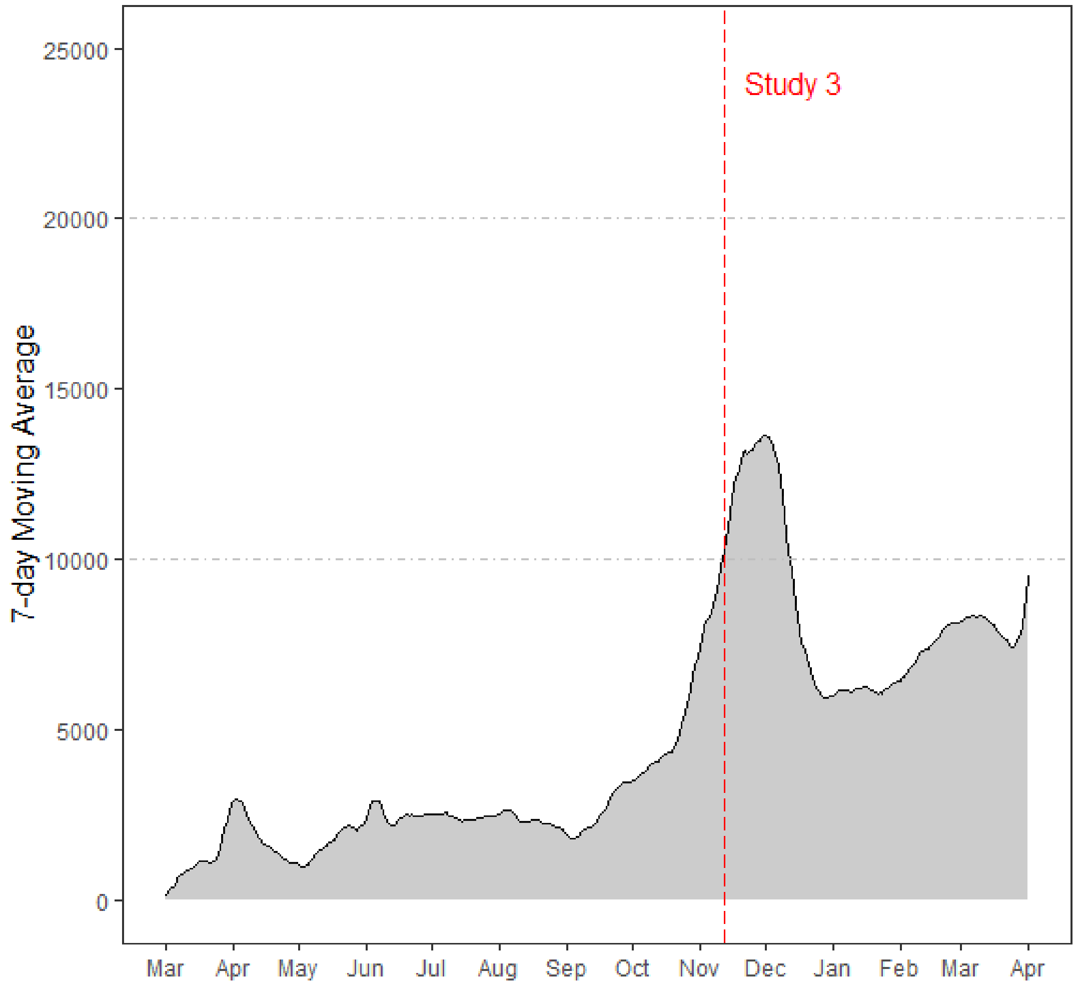

Note. The y-axis represents the 7-day moving average of new COVID-19 cases in Iran. The red line indicates when data collection for Study 2b began.

Factor Analyses

Confirmatory factor analyses (CFAs) were conducted in R 4.0.3. Exploratory factor analyses (EFAs) were conducted in SPSS 22.

Study 1

We conducted CFAs for self-reliance and government distrust as single-factor scales. EFAs suggested 2 factors for COVID-19 behavior: independent action and CDC adherence. Due to low factor loadings, we excluded one COVID-19 behavior item ("take on new sources of income"). See Table 2 below for results.

Based on EFA results from Study 2a, we went back to Study 1 to conduct CFAs on the one-factor and two-factor versions of the Honor Values Scale. The two-factor version showed an improvement from the one-factor version, suggesting better fit.

Study 2a

Emerging civil liberties concerns when we designed and conducted Study 2a (August, September 2020) led us to add a new item to Government Distrust which described the government's inability to "restrain itself from infringing on the rights of individuals" but it showed poor fit with the scale (see Table 2), so we did not include it.

We conducted CFAs for the HVS, self-reliance, government distrust, independent action, and adherence to public health guidelines. Since CFAs for adherence to public health guidelines initially did not converge, we fixed loadings of two items ("stay home as much as possible," "try to convince my loved ones to stay home as much as possible") to be equal, which allowed the models to converge. See Table 2 below for results.

Table 2

Goodness-of-Fit Indicators for Scales (Studies 1-3)

Scale	CFI	TLI	χ^2	*df*	*p*	RMSEA
**Study 1 (U.S.)**						
Self-Reliance	1.00	0.99	5.28	2	0.071	0.065
Government Distrust	0.98	0.97	15.21	5	0.010	0.072
Honor Values Scale (one factor)	0.73	0.70	647.16	153	<0.001	0.098
Honor Values Scale (two factors)	0.83	0.81	454.50	134	<0.001	0.078
**Study 2a (U.S.)**						

Self-Reliance	0.99	0.97	20.03	2	< 0.001	0.124
Independent Action	0.96	0.93	76.14	9	< 0.001	0.113
Government Distrust (without new item)	0.95	0.91	60.40	5	< 0.001	0.138
Government Distrust (with new item)	0.77	0.61	401.91	9	< 0.001	0.273
Honor Values Scale (one factor)	0.78	0.76	888.21	135	< 0.001	0.098
Adherence to Public Health Guidelines	0.99	0.98	10.81	1	0.001	0.129
**Study 2b (Iran)**						
Self-Reliance	0.99	0.96	10.16	2	0.006	0.143
Independent Action	0.90	0.83	56.56	9	< 0.001	0.162
Government Distrust	0.96	0.92	29.83	5	< 0.001	0.157
Adherence to Public Health Guidelines	0.96	0.87	10.52	1	0.001	0.218
Honor Values Scale (one factor)	0.62	0.56	1570.72	153	<0.001	0.142
Honor Values Scale (two factors)	0.73	0.69	521.60	134	<0.001	0.120
**Study 3 (Iran)**						
Moralization of Self-Reliance	1.00	1.02	0.011	1	0.916	0.000
Family Reliance	0.92	0.86	23.84	9	0.005	0.124
Self-Control Moralization	0.76	0.71	331.31	65	< 0.001	0.201
Honor Values Scale (one factor)	0.52	0.46	444.47	135	<0.001	0.146
Honor Values Scale (three factors)	0.86	0.83	159.88	87	<0.001	0.088
**Study 3 (U.S.)**						
Moralization of Self-Reliance	0.99	0.96	1.635	1	0.201	0.073
Family Reliance	0.88	0.80	34.55	9	< 0.001	0.154
Self-Control Moralization	0.89	0.86	218.36	65	< 0.001	0.140
Honor Values Scale (one factor)	0.77	0.74	373.43	135	<0.001	0.121
Honor Values Scale (two factors)	0.84	0.81	302.27	134	<0.001	0.102

Since factor analytic indices for the HVS in Study 2a were low (CFI 0.78, TLI 0.76), we conducted an EFA to examine the factor structure of the scale. EFAs suggested 2 factors for the HVS: one corresponding reputational concerns in honor, and the other corresponding to proper conduct in honor. These factors are consistent with prior theorization about honor. The factor items are shown below in Table 3.

Table 3

Factors of HVS (Study 2a)

Reputation Subscale	Conduct Subscale
It is important for me to keep face in front of others.	I prefer to live with honor, even if it means I will earn less money.
I would jeopardize the honor of my family if I behaved disgracefully.	My word is my bond; is how I feel; it would be dishonorable to behave otherwise.
Reputation matters and should be vigorously defended.	Even if I lost social status if I still have my honor, I can respect myself.
If I am embarrassed, I must not let it show or I will lose face.	Honorable people do not cheat people who trust them.

It is my duty to defend the honor of my family.	I would not disregard my honor even under tough life circumstances.
I would lose face if others saw me misbehave.	Acting right is necessary to maintain my honor.
To maintain my honor, I should not allow myself to be humiliated by others.	Loyalty is a core part of having honor.
My honor will likely be negatively affected if I do not attend to my family obligations.	
My honor depends to a high degree on the appreciation and respect of others.	
Disrespect damages honor.	
I would show I had no honor if I didn't care what others would say.	

Study 2b

We conducted CFAs for the self-reliance, government distrust, independent action, and adherence to public health guidelines scales. Since CFAs for adherence to public health guidelines initially did not converge, we fixed loadings of two items ("stay home as much as possible," "try to convince my loved ones to stay home as much as possible") to be equal, which allowed the models to converge. Based on the Study 2a EFAs for HVS, we also conducted CFAs for both the one-factor and two-factor HVS. The two-factor HVS showed an improvement from the one-factor version, but its factor analytic indices were still low, suggesting that the factor structure of the Iranian HVS might differ from that of the American HVS. Indeed, EFAs suggest a three-factor structure for the Iranian HVS (see Table 4) – one corresponding to reputational concerns (Reputation Subscale), one to acting right (Conduct Subscale), and one to maintaining honor in the face of hardship (Hardship Subscale). Three items were excluded: one for loading onto both Reputation and Hardship ("My honor will likely be negatively affected if I do not attend to my family obligations") and two for low loadings ("To maintain my honor, I should not allow myself to be humiliated by others" and "My word is my bond; is how I feel; it would be dishonorable to behave otherwise").

Table 4

Factors of HVS (Study 2b)

Reputation Subscale	Conduct Subscale	Hardship Subscale
It is important for me to keep face in front of others.	Loyalty is a core part of having honor.	I would not disregard my honor even under tough life circumstances.
I would lose face if others saw me misbehave.	Honorable people do not cheat people who trust them.	I prefer to live with honor, even if it means I will earn less money.
It is my duty to defend the honor of my family.	Acting right is necessary to maintain my honor.	Even if I lost social status if I still have my honor, I can respect myself.
My honor depends to a high degree on the appreciation and respect of others.	Loyalty is a core part of having honor.	
Reputation matters and should be vigorously defended	Honorable people do not cheat people who trust them.	
If I am embarrassed, I must not let it show or I will lose face.	Acting right is necessary to maintain my honor.	
I would jeopardize the honor of my family if I behaved disgracefully.	Loyalty is a core part of having honor.	
I would show I had no honor if I didn't care what others would say.		
Disrespect damages honor.		

Study 3

We conducted CFAs for the HVS, moralization of self-reliance, family reliance, and self-control moralization for both Iran and U.S. data. Since CFAs for adherence to public health guidelines initially did not converge, we fixed loadings of two items ("Relying on others to fulfill my obligations for me," "Asking to be taught instead of learning on my own") to be equal, which allowed the models to converge. Based on the Study 1 and Study 2b EFAs for HVS, we also conducted CFAs for the one-factor and three-factor HVS for Iran and the one-factor and two-factor HVS for the U.S. The three-factor HVS and two-factor HVS showed improvement from the one-factor version.

Other Pre-Registered Analyses

In addition to analyzing public health adherence as an outcome, we also analyzed independent action as an outcome. The scale measures tendencies to act independently of the government (e.g., "seek out protections that make sense to me…", "decide for myself how

serious COVID is"). Based on the idea of honor-as-self-reliance, we had predicted that self-reliance would mediate the relationship between honor and independent action, such that honor would be associated with greater self-reliance and therefore greater independent action. Moreover, drawing on accounts of the origins of honor (Cohen et al., 1996), we reasoned that honor would thrive in environments where governments are untrustworthy. We therefore predicted that government distrust would moderate the effects of honor on self-reliance, strengthening the relationship.

Study 1 (U.S.)

Indirect Effect of Honor on Independent Action through Self-Reliance

Using a mediation model with paths from honor to self-reliance to independent action, we found the predicted indirect effect of honor on independent action through self-reliance, β = 0.13, 95% CI [0.09, 0.19] (bootstrapped 5000 times), mediation effect R^2 = 0.031. Honor value scores no longer related to willingness to take independent action after accounting for preferences for self-reliance, β = 0.08, p = .135.

Since our data were collected cross-sectionally, we conducted an alternate mediation with paths from honor values to independent action to self-reliance. The indirect effect of honor in this direction was significant but smaller, β = 0.05, 95% CI [0.02, 0.09] (bootstrapped 5000 times), suggesting that both paths are possible.

Table 5

Study 1: Results of Linear Regressions Predicting Self-Reliance

Variable	Statistics							
	B [95% CI]	*SE*	*t*	*p*	Adj partial R^2	*F*	*df*	Adj R^2
Honor	0.38 [0.29, 0.47]	0.05	8.20	< .001	0.15	28.41	388	0.17
Distrust	0.09	0.05	1.96	.051	0.01			

	[-0.00, 0.18]				
Honor × Distrust	0.11	0.04	2.59	.010	0.01
	[0.03, 0.20]				

Note: [95% CI]= 95% Confidence Interval, Honor = Honor Values Scale Score, Distrust = Government Distrust Scale Score, Adj = Adjusted

Government Distrust Moderates the Relationship between Honor and Self-Reliance

As detailed in the second row of Table 5 and depicted graphically in Figure 3, linear regression revealed that self-reliance was more strongly related to honor among Americans who distrusted government, β = 0.11, p = .010. As detailed in Figure 4, we also found support for our predictions when we added government distrust as a moderator of the honor values and self-reliance relationship in our mediation model. We found a moderated indirect effect, β = 0.04, 95% CI [0.00, 0.08] (bootstrapped 5000 times). We concretized these results by examining the indirect effect of honor separately for high distrust (≥1 *SD* above mean government distrust) and low distrust participants (≤1 *SD* below mean government distrust). Supporting our prediction, the indirect effect was significant among high-distrust, β = 0.15, 95% CI [0.03, 0.26], but not low-distrust participants, β = 0.01, 95% CI [-0.06, 0.10].

We conducted an alternate mediation with paths to independent action and then self-reliance. The moderated indirect effect was not significant, β = 0.02, 95% CI [-0.01, 0.06] (bootstrapped 5000 times), suggesting that our predicted path may be more robust.

Figure 3

Study 1: Honor Values and Government Distrust Interact to Predict Self-Reliance.

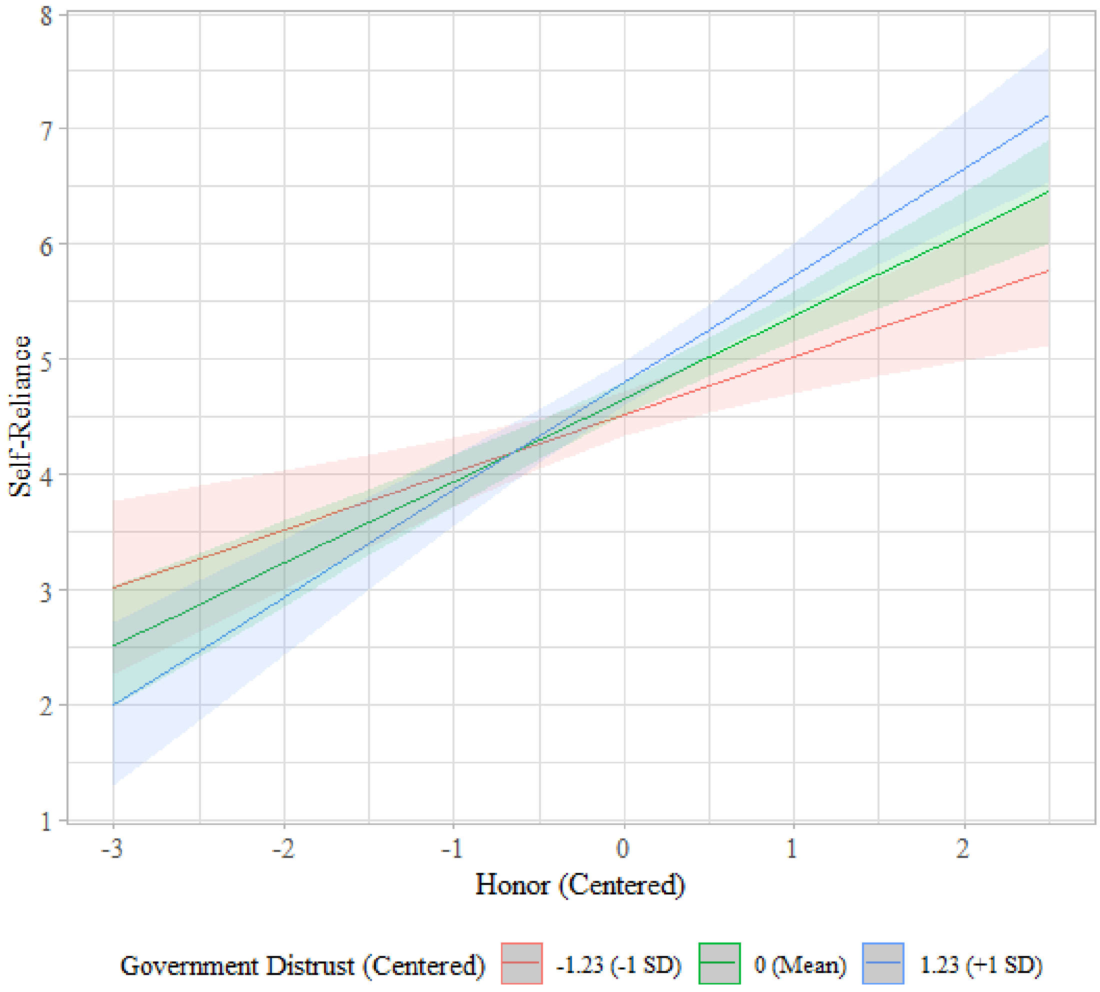

Figure 4

Study 1: The Indirect Effect of Honor on Independent Action through Self-Reliance, Moderated by Government Distrust

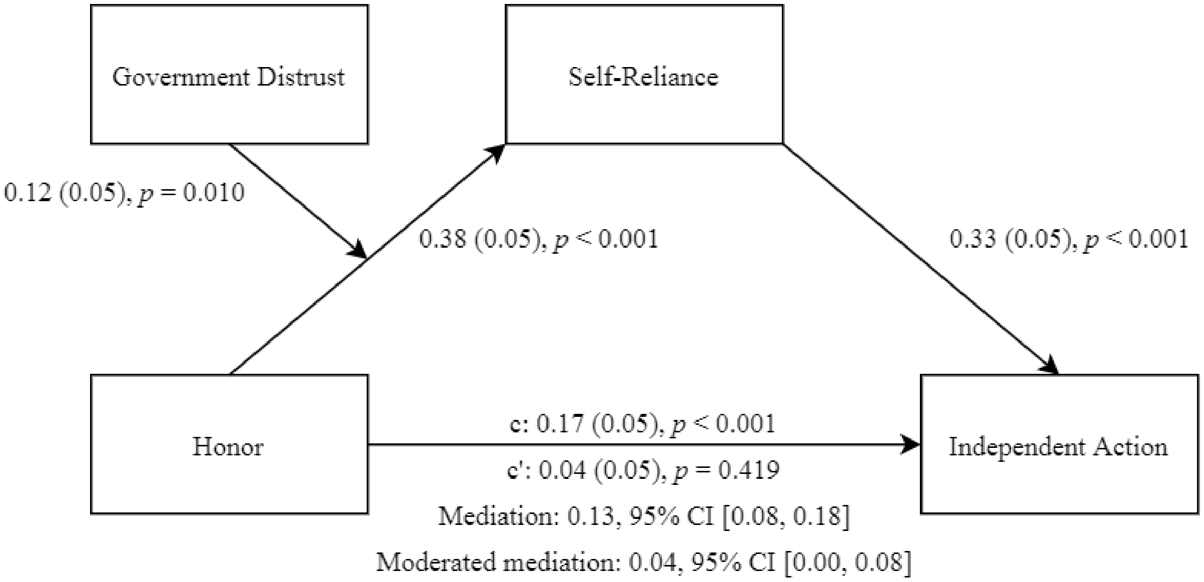

Note. A comprehensive model of our Study 1 results. Standard errors are inside the parentheses.

Study 2a (U.S.)

Indirect Effect of Honor on Independent Action through Self-Reliance

Consistent with Study 1, our mediation model with paths from honor values to self-reliance to independent action revealed an indirect effect of honor on independent action through self-reliance, β = 0.14, 95% CI [0.09, 0.19] (bootstrapped 5000 times), mediation effect R^2 = 0.051. As displayed in Figure 5, the relationship between honor and independent action was smaller in magnitude but still significant after accounting for self-reliance, β = 0.12, p = .001. An alternate model with paths from honor to independent action to self-reliance revealed a similar significant indirect effect of honor, β = 0.13, 95% CI [0.09, 0.18] (bootstrapped 5000 times).

Table 6

Study 2a: Results of Linear Regressions Predicting Self-Reliance

Variable	Statistics							
	β [95% CI]	*SE*	*t*	*p*	Adj partial R^2	*F*	*df*	Adj R^2
Honor	0.25 [0.17, 0.32]	0.04	6.30	< .001	0.06	26.68	582	0.12
Distrust	0.24	0.04	6.05	< .001	0.06			

	[0.16, 0.31]				
Honor × Distrust	-0.05 [-0.12, 0.03]	0.04	1.23	.218	0.00

Note: [95% CI] = 95% Confidence Interval, Honor = Honor Values Scale Score, Distrust = Government Distrust Scale Score, Adj = Adjusted.

Figure 5

Study 2a: Self-Reliance as Mediator Between Honor and Independent Action

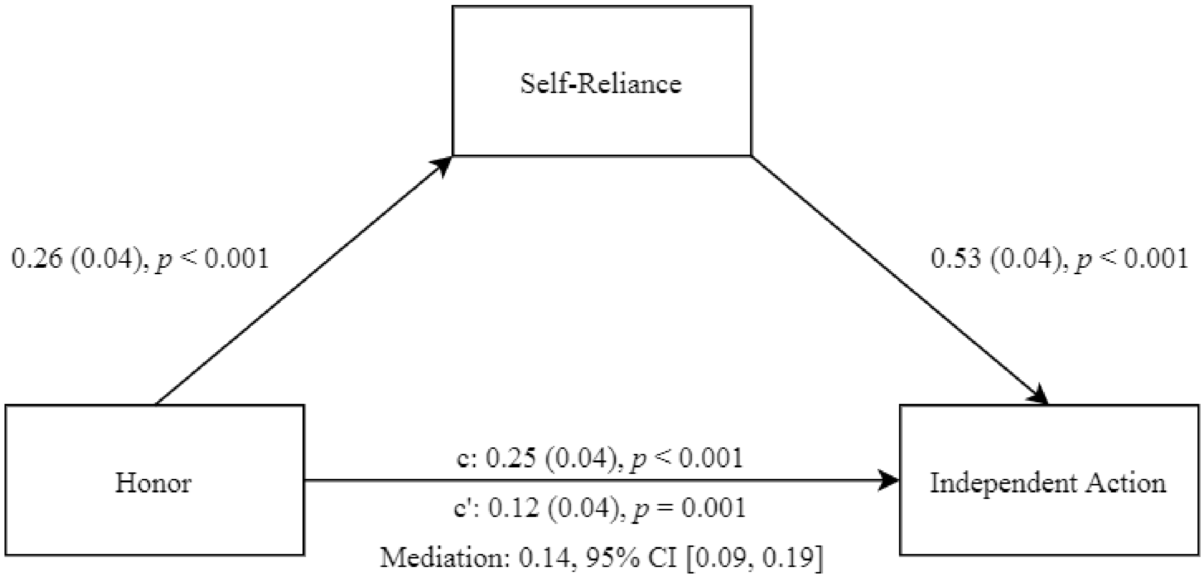

Note. Values inside the parentheses indicate standard errors.

Government Distrust Moderates the Relationship between Honor and Self-Reliance

We did not find that government distrust moderated the relationship between self-reliance and honor, β = -0.05, $p = .218$. These null results are detailed in Table 6.

Study 2b (Iran)

Indirect Effect of Honor on Independent Action through Self-Reliance

As detailed in Table 7, independent action correlated with self-reliance, $r = .39$, $p < .001$, not honor, $r = .06$, $p = .428$. Honor did not correlate with self-reliance, $r = .03$, $p = .692$. Hence, as depicted in Figure 6, there was no indirect effect of honor on independent action through self-reliance, β = 0.01, 95% CI [-0.05, 0.07] (bootstrapped 5000 times), mediation effect $R^2 = 0.001$.

Table 7

Study 2b: Results of Linear Regressions Predicting Self-Reliance

Variable	Statistics							
	β [95% CI]	*SE*	*t*	*p*	Adj partial R^2	*F*	*df*	Adj R^2
Honor	0.05 [-0.09, 0.19]	0.07	0.75	.452	0.00	2.78	197	0.03
Distrust	0.21 [0.06, 0.35]	0.07	2.76	.006	0.03			
Honor × Distrust	-0.10 [-0.22, 0.02]	0.06	1.61	.108	0.01			

Note: [95% CI] = 95% Confidence Interval,
Honor = Honor Values Scale Score, Distrust = Government Distrust Scale Score, Adj = Adjusted.

Figure 6

Study 2b: Self-Reliance as Mediator Between Honor and Independent Action

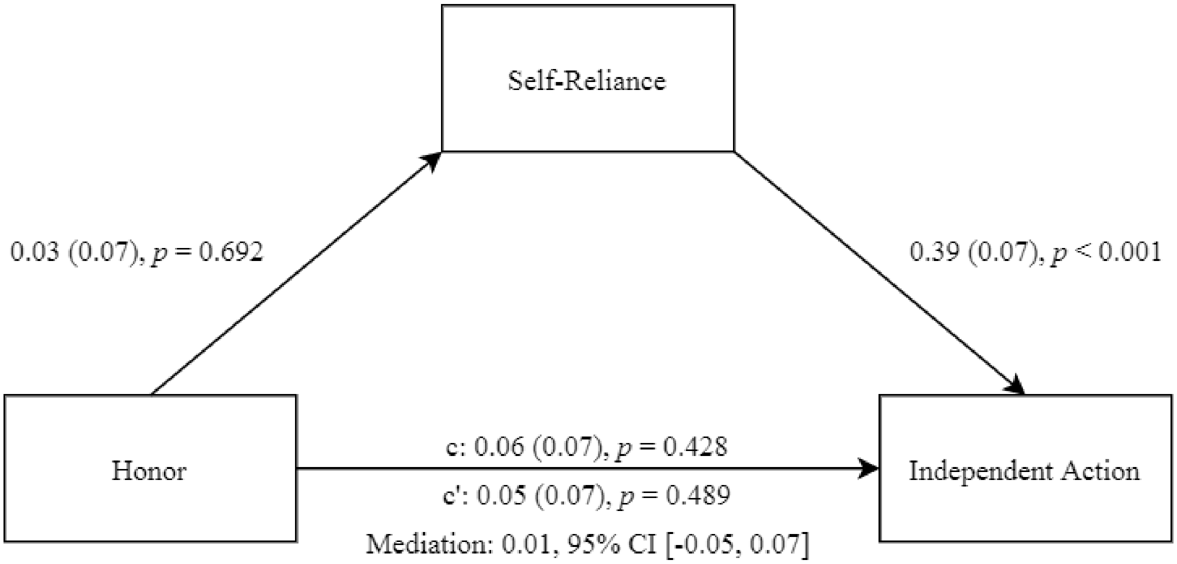

Note. Values inside the parentheses indicate standard errors.

Discussion

Studies 1 and 2a supported our prediction that self-reliance would mediate the honor-independent action relationship. This aligned with our findings on public health adherence.

However, as with public health adherence, Study 2b did not find a mediation of honor and independent action. By using independent action as a separate outcome measure from adherence, we have converging evidence that honor may not operate in Iran the same way it does in the U.S.

Evidence for the moderation of honor and self-reliance by government distrust was mixed. We found evidence for this in Study 1, but not in Study 2a, suggesting that, even within an American context, the findings on government distrust are unstable. This might reflect differences in salient concerns in May 2020 (Study 1) and September 2020 (Study 2a). In May, people were concerned about a lack of general supplies. Shortages, though still existent, were no longer in the news by September, so people may not have linked their distrust in government to response to COVID-19.

Analyses with Honor Subscales

We examined whether the HVS subscales played a similar role in our model as the overall HVS score. We used the two-factor HVS to analyze Studies 1 and 2a. Since factor analyses revealed 3 factors for the HVS in Iran, we analyzed Study 2b using the three-factor HVS.

Study 1

HVS Reputation

Honor is Associated with Self-Reliance. Linear regression revealed that higher scores on the HVS reputation subscale was related to greater preference for self-reliance, $\beta = 0.35$, $p < .001$.

Self-Reliance Mediates the Relationship between Honor and Independent Action. Linear regression revealed that higher reputation scores were related to greater endorsement of independent action, $\beta = 0.21$, $p < .001$. Self-reliance mediated this relationship, $\beta = 0.11$, 95% CI

[0.07, 0.16]; reputation concerns were associated with greater self-reliance, which in turn was associated with greater endorsement of independent action.

Government Distrust Moderates the Relationship between Honor and Self-Reliance. Linear regression predicting self-reliance from reputation concerns and distrust revealed a significant interaction of the two variables, β = 0.14, $p = .001$. The relationship between reputation concerns and self-reliance was stronger among those who reported more distrust in government.

Self-Reliance Mediates the Relationship between Honor and Public Health Adherence. Linear regression revealed no relationship between reputation concerns and public health adherence, β = -0.06, $p = .239$. However, self-reliance mediated the relationship between reputation concerns and public health adherence, β = -0.07, 95% CI [-0.11, -0.03]. Greater reputation concerns were associated with greater self-reliance, which were in turn associated with lower adherence.

HVS Conduct

Honor is Associated with Self-Reliance. Linear regression revealed that higher scores on the HVS conduct subscale was related to greater preference for self-reliance, β = 0.34, $p < .001$.

Self-Reliance Mediates the Relationship between Honor and Independent Action. Linear regression found no significant relationship between conduct scores and independent action, β = 0.07, $p = .186$. However, self-reliance mediated their relationship, β = 0.13, 95% CI [0.08, 0.18]; conduct concerns were associated with greater self-reliance, which in turn was associated with greater endorsement of independent action.

Government Distrust Moderates the Relationship between Honor and Self-Reliance. Linear regression revealed no interaction of conduct scores and independent action, β = 0.07, *p* = .118.

Self-Reliance Mediates the Relationship between Honor and Public Health Adherence. Linear regression revealed no relationship between conduct scores and public health adherence, β = 0.01, *p* = .823. However, self-reliance mediated the relationship between conduct concerns and public health adherence, β = -0.08, 95% CI [-0.12, -0.04]. Greater conduct concerns were associated with greater self-reliance, which were in turn associated with lower adherence.

Discussion

Subscale analyses generally followed the same pattern of results as analyses using total HVS scores. Analyses with both the reputation and conduct subscales supported 3 of our 4 hypotheses: honor is associated with self-reliance, self-reliance mediates the relationship between honor and independent action, and self-reliance mediate the relationship between honor and public health adherence. Government distrust only moderated the relationship between reputation concerns and self-reliance, but did not moderate the relationship between conduct concerns and self-reliance. This is unsurprising, given that reputational aspects of honor are a more central component of our theorization – people who value honor prefer self-reliance in order to show others that they can fend for themselves.

Study 2a

HVS Reputation

Honor is Associated with Self-Reliance. Linear regression revealed that higher scores on the HVS reputation subscale was related to greater preference for self-reliance, $\beta = 0.24$, $p < .001$.

Self-Reliance Mediates the Relationship between Honor and Independent Action. Linear regression revealed that higher reputation scores were related to greater endorsement of independent action, $\beta = 0.21$, $p < .001$. Self-reliance mediated this relationship, $\beta = 0.13$, 95% CI [0.08, 0.18]; reputation concerns were associated with greater self-reliance, which in turn was associated with greater endorsement of independent action.

Government Distrust Moderates the Relationship between Honor and Self-Reliance. Linear regression predicting self-reliance from reputation concerns and distrust revealed no interaction of the two variables, $\beta = -0.05$, $p = .209$.

Self-Reliance Mediates the Relationship between Honor and Public Health Adherence. Linear regression revealed no relationship between reputation concerns and public health adherence, $\beta = 0.01$, $p = .832$. However, self-reliance mediated the relationship between reputation concerns and public health adherence, $\beta = -0.06$, 95% CI [-0.09, -0.04]. Greater reputation concerns were associated with greater self-reliance, which were in turn associated with lower adherence.

HVS Conduct

Honor is Associated with Self-Reliance. Linear regression revealed that higher scores on the HVS conduct subscale was related to greater preference for self-reliance, $\beta = 0.20$, $p < .001$.

Self-Reliance Mediates the Relationship between Honor and Independent Action. Linear regression found that greater conduct concerns were associated with greater endorsement

of independent action, β = 0.24, $p < .001$. Self-reliance mediated this relationship, β = 0.11, 95% CI [0.06, 0.16]; conduct concerns were associated with greater self-reliance, which in turn was associated with greater endorsement of independent action.

Government Distrust Moderates the Relationship between Honor and Self-Reliance. Linear regression revealed no interaction of conduct concerns and independent action, β = -0.03, $p = .486$.

Self-Reliance Mediates the Relationship between Honor and Public Health Adherence. Linear regression revealed no relationship between conduct concerns and public health adherence, β = -0.07, $p = .115$. However, self-reliance mediated the relationship between conduct concerns and public health adherence, β = -0.05, 95% CI [-0.07, -0.02]. Greater conduct concerns were associated with greater self-reliance, which were in turn associated with lower adherence.

Discussion

As in Study 1, subscale analyses in Study 2a followed the same pattern of results as analyses using total HVS scores. Analyses with both the reputation and conduct subscales supported 3 of our 4 hypotheses: honor is associated with self-reliance, self-reliance mediates the relationship between honor and independent action, and self-reliance mediate the relationship between honor and public health adherence. Government distrust did not moderate the relationship between reputation concerns and self-reliance, or between conduct concerns and self-reliance, mirroring our findings with the total HVS scores.

Study 2b

HVS Reputation

Honor is Associated with Self-Reliance. Linear regression revealed no relationship between the HVS reputation subscale and self-reliance, β = 0.08, *p* = .262.

Self-Reliance Mediates the Relationship between Honor and Independent Action. Linear regression revealed no relationship between reputation concerns and independent action, β = 0.05, *p* = .440. Self-reliance did not mediate this relationship, β = 0.03, 95% CI [-0.03, 0.09].

Government Distrust Moderates the Relationship between Honor and Self-Reliance. Linear regression predicting self-reliance from reputation concerns and distrust revealed no interaction of the two variables, β = -0.11, *p* = .087.

Self-Reliance Mediates the Relationship between Honor and Public Health Adherence. Linear regression revealed no relationship between reputation concerns and public health adherence, β = 0.08, *p* = .282. Self-reliance did not mediate the relationship between reputation concerns and public health adherence, β = -0.02, 95% CI [-0.07, 0.02].

HVS Conduct

Honor is Associated with Self-Reliance. Linear regression revealed no relationship between the conduct subscale and self-reliance, β = -0.04, *p* = .608.

Self-Reliance Mediates the Relationship between Honor and Independent Action. Linear regression found no association between conduct concerns and endorsement of independent action, β = 0.05, *p* = .518. Self-reliance did not mediate this relationship, β = -0.01, 95% CI [-0.08, 0.04].

Government Distrust Moderates the Relationship between Honor and Self-Reliance. Linear regression revealed no interaction of conduct concerns and independent action, β = -0.08, *p* = .247.

Self-Reliance Mediates the Relationship between Honor and Public Health Adherence. Linear regression revealed that greater conduct concerns were associated with greater endorsement of adherence to public health guidelines, β = 0.27, $p < .001$. Self-reliance did not mediate this relationship, β = 0.01, 95% CI [-0.03, 0.05].

HVS Hardship

Honor is Associated with Self-Reliance. Linear regression revealed no relationship between the hardship subscale and self-reliance, β = -0.09, $p = .216$.

Self-Reliance Mediates the Relationship between Honor and Independent Action. Linear regression found no association between the hardship subscale and endorsement of independent action, β = 0.05, $p = .475$. Self-reliance did not mediate this relationship, β = -0.03, 95% CI [-0.11, 0.03].

Government Distrust Moderates the Relationship between Honor and Self-Reliance. Linear regression revealed no interaction of the hardship subscale and independent action, β = -0.06, $p = .359$.

Self-Reliance Mediates the Relationship between Honor and Public Health Adherence. Linear regression revealed that greater hardship subscale scores were associated with greater endorsement of adherence to public health guidelines, β = 0.20, $p = .004$. Self-reliance did not mediate this relationship, β = 0.02, 95% CI [-0.02, 0.07].

Discussion

Mirroring analyses with the total HVS scores, our subscale analyses for Study 2b did not support any of our 4 hypotheses. Moreover, as with the total HVS scores, higher scores on the hardship and conduct subscales were associated with greater adherence to public health guidelines. Since the hardship ("I would not disregard my honor even under tough life

circumstances") items emphasize maintaining honor in the face of challenges, the subscale may motivate attentiveness towards public health guidelines during the challenging times of the pandemic. Since the conduct ("Acting right is necessary to maintain my honor") items emphasize virtuous action, the subscale may also motivate adherence to public health guidelines, given that public health adherence can protect others in the community.

Study 3 – Iran

HVS Reputation

Regression predicting Reputation scores from moralization of self-reliance and family reliance revealed a significant relationship between Reputation and family reliance, $\beta = 0.23$, $p = .017$, but no relationship with moralization of self-reliance, $\beta = 0.08$, $p = .382$.

HVS Conduct

Regression predicting Conduct scores from moralization of self-reliance and family reliance revealed a significant relationship between Conduct and family reliance, $\beta = 0.40$, $p < .001$, but no relationship with moralization of self-reliance, $\beta = 0.15$, $p = .088$.

HVS Hardship

Regression predicting Hardship scores from moralization of self-reliance and family reliance revealed a significant relationship between Hardship and family reliance, $\beta = 0.20$, $p = .042$, but no relationship with moralization of self-reliance, $\beta = 0.00$, $p = .999$.

Discussion

Our subscales in Study 3 followed the same pattern of results as the total HVS scores. Family reliance predicted Reputation, Conduct, and Hardship but moralization of self-reliance did not.

Study 3 – U.S.

HVS Reputation

Regression predicting Reputation scores from moralization of self-reliance and family reliance revealed a significant relationship between Reputation and family reliance, β = 0.47, $p < .001$, and with moralization of self-reliance, β = 0.27, $p < .001$.

HVS Conduct

Regression predicting Conduct scores from moralization of self-reliance and family reliance revealed a significant relationship between Conduct and family reliance, β = 0.39, $p < .001$, and with moralization of self-reliance, β = 0.21, $p = .012$.

Discussion

Our subscales in Study 3 followed the same pattern of results as the total HVS scores. Family reliance and moralization of self-reliance predicted Reputation and Conduct.

Measurement Invariance

Our studies were conducted in the U.S. and Iran to measure the relationships among honor, self-reliance, independent action, public health adherence, and government distrust. We therefore conducted measurement invariance analyses for our key variables, comparing responses from the U.S. and Iran. Items for honor, self-reliance, and government distrust were the same across all 3 studies, so we used data from the 3 studies to examine measurement invariance for those constructs. Items for independent action and public health adherence were changed after Study 1, so we only used data from Studies 2a and 2b to examine measurement invariance for those constructs.

Honor

Measurement invariance test results for the Honor Values Scale as one factor are shown in Table 8 below. Low model fit indices (CFI = 0.738) indicate configural noninvariance,

suggesting that the factor structure of honor varied between the U.S. and Iran. Model fit indices for the HVS as two factors (CFI = 0.826) showed an improvement, but were still low, again suggesting that the U.S. and Iranian samples did not share a common factor structure for honor. Since results revealed configural noninvariance, we did not proceed further in invariance decisions.

Configural noninvariance of honor is consistent with prior work suggesting that honor in Iran is more multifaceted than and organized differently from honor in the U.S. (Atari et al., 2020). This finding also aligns with results from the main report – since honor is structured differently in Iran than in the U.S., it may not hold the same emphasis on self-reliance.

Table 8

Measurement Invariance Test for Honor (One Factor)

Model	χ^2 (*df*)	CFI	RMSEA	Model compared	$\Delta\chi^2$ (Δdf)	*p*	ΔCFI	ΔRMSEA
M1: Configural Invariance	2051.72 (270)	0.738	0.106	-	-	-	-	-
M2: Metric Invariance	2141.22 (287)	0.727	0.105	M1	89.50 (17)	< .001	0.011	0.001
M3: Scalar Invariance	2804.31 (304)	0.632	0.118	M2	663.09 (17)	< .001	0.095	0.013
M4: Residual Invariance	2869.29 (305)	0.623	0.119	M3	64.98 (1)	< .001	0.009	0.001

Self-Reliance

Measurement invariance test results for the self-reliance scale are shown in Table 9 below. Good model fit indices (CFI = 0.993) indicate configural invariance, suggesting that the U.S. and Iranian samples shared a common factor structure for self-reliance. We then examined metric invariance. For our invariance analyses, we used the criterion of a less than 0.01 change in CFI, based on concerns that the χ^2 criterion may be overly conservative (Putnick & Bornstein, 2016).

Results revealed metric invariance as well, with a 0.000 change in CFI from the configural invariance model, indicating similar item loadings onto the self-reliance factor across our samples. We did not find scalar invariance (0.013 change in CFI from the metric invariance model), suggesting that item intercepts were not equivalent across our samples. Since results revealed scalar noninvariance, we did not proceed further in invariance decisions.

Our analyses indicate that Americans and Iranians understood our self-reliance items in similar ways – both the factor structure of self-reliance and the loading of items onto this structure were similar across the samples.

Table 9

Measurement Invariance Test for Self-Reliance

**Model**	**χ^2 (*df*)**	**CFI**	**RMSEA**	**Model compared**	**$\Delta\chi^2$ (Δdf)**	***p***	**ΔCFI**	**ΔRMSEA**
M1: Configural Invariance	28.81 (4)	0.993	0.103	-	-	-	-	-
M2: Metric Invariance	31.36 (7)	0.993	0.077	M1	2.44 (3)	.486	0.000	0.026
M3: Scalar Invariance	83.35 (10)	0.980	0.112	M2	52.09 (3)	< .001	0.013	0.035
M4: Residual Invariance	282.73 (11)	0.926	0.205	M3	199.39 (1)	< .001	0.054	0.093

Independent Action

Measurement invariance test results for the independent action scale are shown in Table 10 below. Model fit indices (CFI = 0.943) indicate configural invariance, suggesting that the U.S. and Iranian samples shared a common factor structure for independent action. We then examined metric invariance. Results revealed metric invariance as well, with a 0.007 change in CFI from the configural invariance model, indicating similar item loadings onto the independent action factor across our samples. We did not find scalar invariance (0.048 change in CFI from the metric invariance

model), suggesting that item intercepts were not equivalent across our samples. Since results revealed scalar noninvariance, we did not proceed further in invariance decisions.

Our analyses indicate that Americans and Iranians understood our independent action items in similar ways – both the factor structure of independent action and the loading of items onto this structure were similar across the samples.

Table 10

Measurement Invariance Test for Independent Action

Model	χ^2 (*df*)	CFI	RMSEA	Model compared	$\Delta\chi^2$ (Δdf)	*p*	ΔCFI	ΔRMSEA
M1: Configural Invariance	132.07 (18)	0.943	0.127	-	-	-	-	-
M2: Metric Invariance	151.42 (23)	0.936	0.119	M1	19.35 (5)	.002	0.007	0.008
M3: Scalar Invariance	253.95 (28)	0.888	0.143	M2	102.53 (5)	< .001	0.048	0.024
M4: Residual Invariance	256.70 (29)	0.887	0.141	M3	2.75 (1)	.097	0.001	0.002

Public Health Adherence

Model fit indices (CFI = 0.992) indicate configural invariance, suggesting that the U.S. and Iranian samples shared a common factor structure for public health adherence. However, due to inadequate degrees of freedom, we were unable to progress with the invariance analyses.

Government Distrust

Measurement invariance test results for the government distrust scale are shown in Table 11 below. Model fit indices (CFI = 0.968) indicate configural invariance, suggesting that the U.S. and Iranian samples shared a common factor structure for government distrust. We then examined metric invariance. Results revealed metric invariance as well, with a 0.004 change in CFI from the configural invariance model, indicating similar item loadings onto the government distrust factor across our samples. We did not find scalar invariance (0.027 change in CFI from the metric invariance

model), suggesting that item intercepts were not equivalent across our samples. Since results revealed scalar noninvariance, we did not proceed further in invariance decisions.

Our analyses indicate that Americans and Iranians understood our government distrust items in similar ways – both the factor structure of government distrust and the loading of items onto this structure were similar across the samples.

Table 11

Measurement Invariance Test for Government Distrust

Model	χ^2 (*df*)	CFI	RMSEA	Model compared	$\Delta\chi^2$ (Δdf)	*p*	ΔCFI	ΔRMSEA
M1: Configural Invariance	87.71 (10)	0.968	0.115	-	-	-	-	-
M2: Metric Invariance	101.23 (14)	0.965	0.103	M1	13.52 (4)	.009	0.004	0.012
M3: Scalar Invariance	171.36 (18)	0.938	0.120	M2	70.13 (4)	< .001	0.027	0.017
M4: Residual Invariance	201.21 (19)	0.926	0.128	M3	29.84 (1)	< .001	0.012	0.007

Regional Differences

Since prior literature has documented regional differences within the U.S. in honor culture (Cohen et al., 1996), we tested for differences on our key variables between southern and non-southern states, as well as between red and blue states for exploratory purposes.

Study 1

U.S. South vs. Other States

We conducted independent samples t-tests on each of our key variables to examine differences between southern and non-southern states. Applying Bonferroni correction for 10 tests, we tested each difference at $\alpha = 0.05/10 = 0.005$. There were no differences on mean endorsement of self-reliance, Welch-corrected $t(277.63) = 0.07$, $p = .948$, honor, Welch-

corrected *t*(286.86) = 0.68, *p* = .494, government distrust, Welch-corrected *t*(283.21) = 1.48, *p* = .140, independent action, Welch-corrected *t*(300.71) = 0.28, *p* = .781, or public health adherence, Welch-corrected *t*(267.18) = 1.15, *p* = .250. Since our sample did not capture regional differences in our key constructs, we did not proceed further with regional analyses.

Red vs. Blue States

We conducted independent samples t-tests on each of our key variables to examine differences between red and blue states. Applying Bonferroni correction for 10 tests, we tested each difference at α = 0.05/10 = 0.005. There were no differences on mean endorsement of self-reliance, Welch-corrected *t*(355.09) = 1.24, *p* = .217, honor, Welch-corrected *t*(368.81) = 0.88, *p* = .380, government distrust, Welch-corrected *t*(377.65) = 0.35, *p* = .727, independent action, Welch-corrected *t*(361.60) = 0.08, *p* = .938, or public health adherence, Welch-corrected *t*(332.07) = 1.58, *p* = .115. Since our sample did not capture regional differences in our key constructs, we did not proceed further with regional analyses.

Study 2a

U.S. South vs. Other States

Applying Bonferroni correction for 10 tests, we tested each difference at α = 0.05/10 = 0.005. Participants from southern states expressed greater endorsement of self-reliance, Welch-corrected *t*(409.35) = 3.16, *p* = .002, Cohen's *d* = 0.27, compared to participants from non-southern states. There were no differences in mean endorsement on honor, Welch-corrected *t*(444.64) = 2.13, *p* = .034, government distrust, Welch-corrected *t*(373.36) = 0.54, *p* = .587, independent action, Welch-corrected *t*(400.59) = 2.33, *p* = .020, and adherence, Welch-corrected *t*(332.81) = 1.98, *p* = .049, between the two regions. Means are shown below in Table 12.

Table 12

Mean Endorsement of Key Variables, Southern vs. Non-southern States (Study 2a)

**Variable**	**Southern States**	**Non-southern States**
	M (*SD*)	*M* (*SD*)
Self-reliance	4.88 (1.59)	4.43 (1.70)
Honor	4.86 (0.77)	4.71 (0.90)
Government distrust	4.78 (1.33)	4.84 (1.29)
Independent action	4.74 (1.29)	4.99 (1.24)
Public health adherence	6.19 (1.08)	5.98 (1.28)

We then examined whether our hypothesized relationships between variables differed between regions.

Honor is Associated with Self-Reliance. Linear regression predicting self-reliance from the interaction of honor scores and region revealed no significant interaction, β = -0.03, *p* = .767. However, honor scores did significantly predict self-reliance, β = 0.25, *p* < .001.

Self-Reliance Mediates the Relationship between Honor and Independent Action. Linear regression predicting independent action from the interaction of honor scores and region revealed no significant interaction, β = 0.09, *p* = .346. While self-reliance mediated honor and independent action, β = 0.13, 95% CI [0.08, 0.18], region did not moderate this mediation, β = -0.01, 95% CI [-0.05, 0.04].

Government Distrust Moderates the Relationship between Honor and Self-Reliance. Linear regression predicting self-reliance from the interaction of honor, government distrust, and region revealed no significant interaction of the three variables, β = -0.00, *p* = .993. The interaction of honor and government distrust was also not significant, β = -0.05, *p* = .269.

Self-Reliance Mediates the Relationship between Honor and Public Health Adherence. Linear regression predicting public health adherence from the interaction of honor and region revealed no significant interaction, β = -0.13, *p* = .159. There was also no significant main effect of honor, β = 0.02, *p* = .639. While self-reliance mediated the relationship between

honor and public health adherence, β = -0.06, 95% CI [-0.09, -0.04], region did not moderate this mediation, β = 0.00, 95% CI [-0.02, 0.03].

Discussion. Study 2a showed a difference between southern and non-southern states in mean endorsement of self-reliance. Investigating further, we found no evidence that being in a southern state changed the pattern of relationships between our key constructs. Our findings from the main report hold regardless of whether participants come from the South or a different region.

Red vs. Blue States

We conducted independent samples t-tests on each of our key variables to examine differences between red and blue states. Applying Bonferroni correction for 10 tests, we tested each difference at $\alpha = 0.05/10 = 0.005$. There were no differences on mean endorsement of self-reliance, Welch-corrected $t(510.99) = 2.35$, $p = .019$, honor, Welch-corrected $t(525.32) = 0.00$, $p = .998$, government distrust, Welch-corrected $t(473.83) = 0.06$, $p = .954$, independent action, Welch-corrected $t(503.47) = 1.43$, $p = .152$, or public health adherence, Welch-corrected $t(407.03) = 1.55$, $p = .122$. Since our sample did not capture regional differences in our key constructs, we did not proceed further with regional analyses.

Other Pre-Registered Mediation Analyses Controlling for Secondary Variables

Analyses were conducted in R 4.0.3.

Study 1

In our moderated mediation we drew paths from honor to self-reliance (*a*-path) and from self-reliance to independent action with government distrust moderating the relationship between honor and self-reliance. We controlled for both political orientation and gender in our *a*-path from honor to self-reliance in our moderated-mediation model. We did so given the literature

which suggests that honor culture in the U.S. is geographically concentrated in the South (Cohen et al., 1996), where conservatism is deeply-rooted (Phillips-Fein, 2011), and that honor-based norms are sometimes specified for a particular gender (Cohen et al., 1996). We scored political orientation by averaging orientations in the social and economic domains ($\alpha = 0.86$). Six participants identified as "other" on the gender item and 1 person did not respond to the gender item; our analyses include the 217 male and 168 female responding participants for a total sample size of 385. We coded male as 0 and female as 1.

As predicted, the mediation of honor and independent action by self-reliance remained significant after controlling for political orientation and gender (path *a*, $\beta = 0.09$, 95% CI [0.05, 0.14]). This was a full mediation, such that the relationship between honor and independent action, $\beta = 0.10$, $p = .041$, was no longer significant after accounting for self-reliance, $\beta = 0.01$, $p = .844$. The interaction term of honor and government distrust followed the same pattern in the main report, significantly predicting self-reliance, $\beta = 0.11$, $p = .013$, such that the relationship between honor and self-reliance was stronger among those higher on government distrust. However, the moderated mediation effect was no longer significant, $\beta = 0.03$, 95% CI [-0.00, 0.07]. The model is shown below in Fig. 3. We obtained mediation effect estimates after bootstrapping 5,000 times.

Additionally, self-reliance mediated the relationship between political orientation and independent action, $\beta = 0.08$, 95% CI [0.05, 0.12]; conservatism predicted greater self-reliance, $\beta = 0.28$, $p < .001$, and self-reliance in turn predicted greater independent action, $\beta = 0.29$, $p < .001$. This was a partial mediation, such that the relationship between political orientation and independent action, $\beta = 0.19$, $p < .001$, was reduced in magnitude but still significant after

accounting for self-reliance, β = 0.11, p = .034. Gender did not predict self-reliance, β = 0.02, p = .723, and hence there was no mediation of gender and independent action by self-reliance.

Figure 7

Mediation of Honor and Independent Action by Self-Reliance, Moderated by Government Distrust (Study 1)

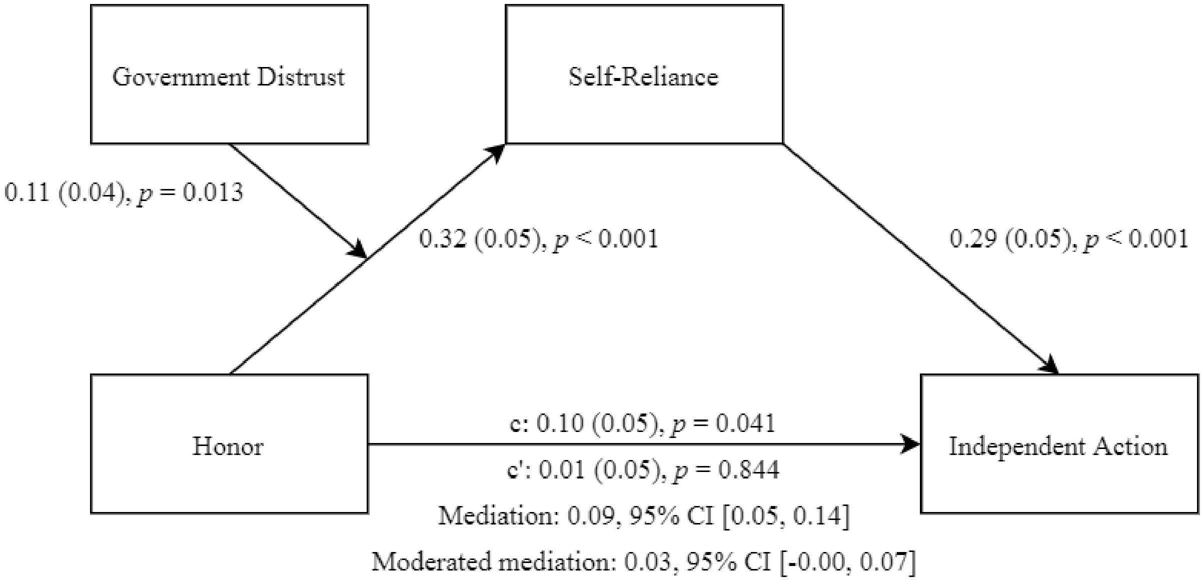

Note. After we controlled for political orientation and gender, self-reliance still mediated honor and independent action. The relationship between honor and self-reliance (path *a*) was moderated by government distrust.

Study 2a

As in Study 1, in the mediation model, we controlled for political orientation (α = 0.88) and gender (path *a*) drawing paths from honor to self-reliance to independent action. We excluded 8 participants who identified as “other” on the gender item for a total of 578 participants (256 males, 322 females). We coded males as 0 and females as 1.

As predicted, the mediation of honor and independent action by self-reliance remained significant after controlling for political orientation and gender (path *a*, β = 0.08, 95% CI [0.03,

0.13]). This was a partial mediation, such that the relationship between honor and independent action, β = 0.16, $p < .001$, was reduced in magnitude but still significant after accounting for self-reliance, β = 0.08, $p = .033$. The model is shown below in Fig. 4. We obtained mediation effect estimates after bootstrapping 5,000 times.

Additionally, self-reliance mediated the relationship between political orientation and independent action, β = 0.14, 95% CI [0.10, 0.18]; conservatism predicted greater self-reliance, β = 0.27, $p < .001$, and self-reliance in turn predicted greater independent action, β = 0.50, $p < .001$. This was a partial mediation, such that the relationship between political orientation and independent action, β = 0.27, $p < .001$, was reduced in magnitude but still significant after accounting for self-reliance, β = 0.13, $p < .001$. Gender did not predict self-reliance, β = 0.01, $p = .758$, and hence there was no mediation of gender and independent action by self-reliance.

Figure 8

Mediation of Honor and Independent Action by Self-Reliance (Study 2a)

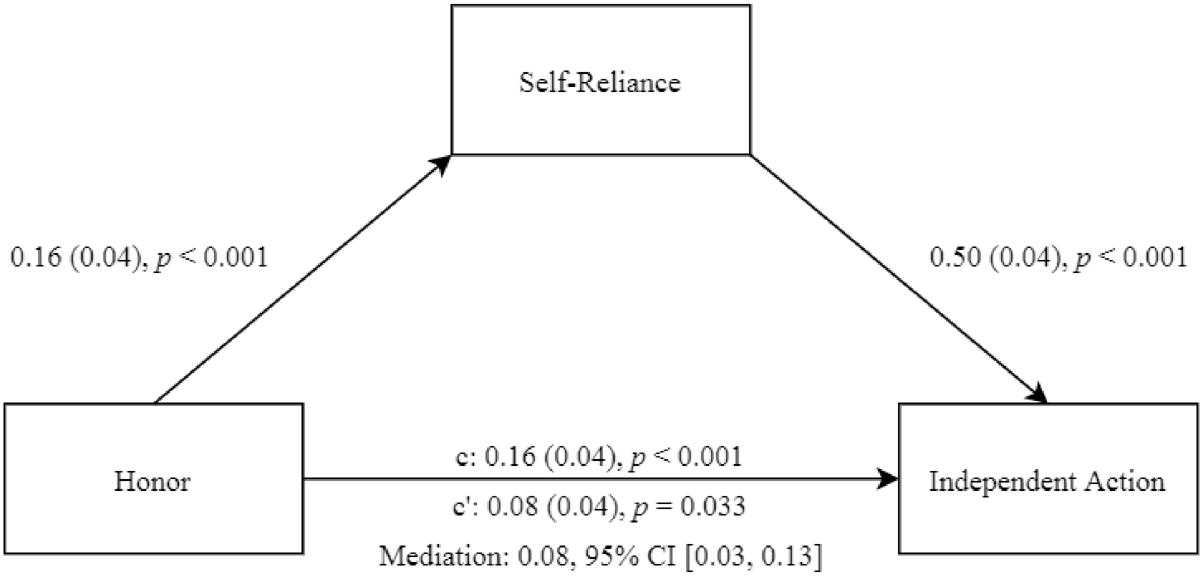

Note. After we controlled for political orientation and gender, self-reliance still mediated the relationship between honor and independent action.

Study 2b

Analyses from the main report showed that self-reliance did not mediate honor and independent action in Iran. Since the sample of 201 participants was mostly left-leaning in political orientation (85.1%), we controlled for political orientation and the interaction of political orientation with honor in the mediation model (path *a*).

We still did not find a significant mediation of honor and independent action by self-reliance, β = -0.00, 95% CI [-0.07, 0.06], since honor did not predict self-reliance, β = -0.01, p = .928. Moreover, political orientation did not moderate this relationship, β = -0.07, p = .423, nor did it moderate the mediation of honor and independent action by self-reliance, β = -0.03, 95% CI [-0.10, 0.05]. This is consistent with the null results from the main report and suggests that our pattern of results did not differ between left-leaning and right-leaning participants. The model is shown below in Fig. 5. We obtained mediation effect estimates after bootstrapping 5,000 times.

Self-reliance did not mediate the relationship between political orientation and independent action, β = 0.04, 95% CI [-0.02, 0.10], since political orientation did not predict self-reliance, β = 0.10, p = .175.

Figure 9

Mediation of Honor and Independent Action by Self-Reliance (Study 2b)

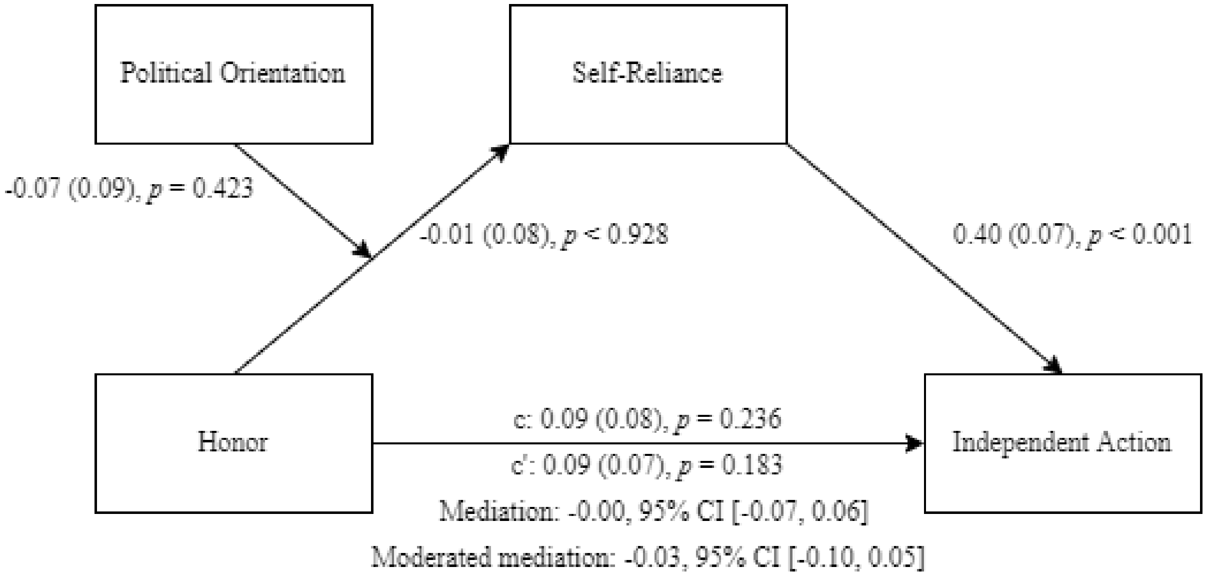

Note. After we controlled for political orientation, the mediation effect was still not significant.

Discussion

As we predicted, controlling for political orientation and gender in the U.S. samples (Studies 1 and 2a) did not change our pattern of results. Self-reliance still mediated the relationship between honor and independent action, demonstrating that our findings in the U.S. samples were robust to the inclusion of variables theoretically related to honor. Consistent with results from the main report, controlling for political orientation in Iran (Study 2b) did not change our pattern of results either – there was still no significant mediation effect, nor did the mediation differ by political orientation.

Exploratory Analyses with Order of Scales

We randomly assigned the order of the HVS in Study 1 and Study 2a to before or after other scales to check if the accessibility of honor values would influence self-reliance. When the HVS appears first, honor values may be more accessible when completing other scales. Hence the accessibility of honor values may influence the endorsement of self-reliance. Aligning with our predictions from the main report, we also checked if this relationship would be stronger

among people higher on government distrust, in moderation analyses. Analyses were conducted in 4.0.3.

Study 1

Participants either saw portions of the HVS before each of the other scales, or they saw the entire HVS after all the other scales were presented. We conducted analyses on the 392 participants who met inclusion and data quality check criteria.

We found no influence of HVS order on self-reliance in a linear regression, $\beta = -0.03$, $p = .596$. Moreover, government distrust did not interact with HVS order to influence self-reliance in a linear regression, $\beta = -0.06$, $p = .168$.

Study 2a

Participants saw the entire HVS either before the other scales or after the other scales were presented. We conducted analyses on the 586 participants who met inclusion and data quality check criteria.

We found no influence of HVS order on self-reliance in a linear regression, $\beta = 0.04$, $p = .280$. Moreover, government distrust did not interact with HVS order to influence self-reliance in a linear regression, $\beta = 0.02$, $p = .495$.

Discussion

Results from Studies 1 and 2a suggest that manipulating accessibility of honor values by randomly assigning HVS order did not influence endorsement of self-reliance, nor did it interact with government distrust to influence endorsement of self-reliance.

References

Atari, M., Graham, J., & Dehghani, M. (2020). Foundations of morality in Iran. *Evolution and Human Behavior*, *41*(5), 367-384.

Cohen, D., Nisbett, R. E., Bowdle, B. F., & Schwarz, N. (1996). Insult, aggression, and the southern culture of honor: An "experimental ethnography". *Journal of Personality and Social Psychology*, *70*(5), 945-960.

Dong, E., Du, H., & Gardner, L. (2020). An interactive web-based dashboard to track COVID-19 in real time. *The Lancet. Infectious Diseases*, *20*(5), 533-534. https://doi.org/10.1016/S1473-3099(20)30120-1

Novin, S., & Oyserman, D. (2016). Honor as cultural mindset: Activated honor mindset affects subsequent judgment and attention in mindset-congruent ways. *Frontiers in Psychology*, *7*, 1921.

Phillips-Fein, K. (2011). Conservatism: A state of the field. *The Journal of American History*, *98*(3), 723-743.

Putnick, D. L., & Bornstein, M. H. (2016). Measurement invariance conventions and reporting: The state of the art and future directions for psychological research. *Developmental Review*, *41*, 71-90.

Ritchie, H., Ortiz-Ospina, E., Beltekian, D., Mathieu, E., Hasell, J., Macdonald, B., Giattino, C., Appel, C., Rodés-Guirao, L., & Roser, M. (2020). *Coronavirus pandemic (COVID-19)*. OurWorldInData.org. https://ourworldindata.org/coronavirus
