# Supplementary material for: Who can I count on: Honor, self-reliance, and family in the United States and Iran
Source: PLoS One. 2024 Aug 27;19(8):e0306460. doi: 10.1371/journal.pone.0306460 (PMC11349206; doi:10.1371/journal.pone.0306460)
Supplement: Attachment — Submitted filename: response-to-reviewers.pdf [file pone.0306460.s003.pdf]

May 24, 2024

Dear Dr. Banerjee,

Thank you for the opportunity to revise and resubmit our manuscript, *Who Can I Count On: Honor, Self-Reliance, and Family in the United States and Iran*. We greatly appreciate your insights, which have been very helpful in improving our manuscript. This letter describes how we addressed the reviewers' feedback. For clarity, we have organized our specific responses to each of your points in the table on the following page. In the revised manuscript, we have kept track-changes on while removing author information, so you can see the exact changes we made.

Overall, we thank the reviewers for their positive and helpful feedback. Reviewer 1 noted issues with the clarity of our paper, and we have incorporated their suggestions in the manuscript. In particular, we made changes to the introduction to more directly align its discussion with our main ideas and elaborate on our understanding of self-reliance. Reviewer 2 generally noted methodological limitations and reminded us that there is substantial cultural heterogeneity in Iran as well, and we have made changes in response to their feedback, particularly in the discussion section. Both Reviewers noted the age difference between our U.S. and Iranian samples, so we re-ran our analyses controlling for age, as well as other demographic variables such as gender and political affiliation.

We think that all of your points were reasonable critiques and have worked to address them in our paper. As a result, we believe that our paper is now clearer in its conceptualization, and we hope we have eased some concerns about the limitations of our studies. Thank you again for the opportunity to revise our paper. We hope that you and the reviewers agree that it is now ready for publication in PLOS ONE.

In addition, we would like to confirm that this work was supported by the Mind and Society Center, University of Southern California, Los Angeles, CA, and the Department of Psychology, University of Southern California, Los Angeles, CA. The funders had no role in study design, data collection and analysis, decision to publish, or preparation of the manuscript. We also moved the ethics statement to the Methods section, selected a new data repository, uploaded the questionnaire on inclusivity, and revised formatting to meet the standards of PLOS ONE.

Thank you for your consideration of our revisions.

Sincerely,

Peter Wang, Ph.D. (on behalf of all authors)

	Reviewer Feedback	Authors' Response
Reviewer 1	Writing of the introduction can be improved for clarity. The link between the ideas is not established well and paragraphs lack coherence.	We thank Reviewer 1 for their helpful feedback. We have revised the introduction to focus more directly on the research question at hand and avoid discussion of tangential constructs (pages 2-4). We have also elaborated further on certain points in the introduction to make our conceptualization clearer (pages 4, 6, 7, 12).
	In their operationalization of self-reliance, authors seem to contrast self-reliance with reliance in the government. This is evidenced by their self-reliance measure: "During this pandemic, instead of relying on official sources, I should trust myself" These two, however, may not be mutually exclusive. One can, for example, have high levels of trust on themselves AND the government. In other words, self-reliance is operationalized to show disobedience to government. Then it is shown to predict lower levels of public health adherence.	Thank you for this suggestion. We considered self-reliance in opposition to dependence on government partly because this has been an important theme in honor research and because governments were a major source of pandemic-related guidelines. To make our understanding of self-reliance explicit, we elaborated further on self-reliance values in the introduction and methods sections (pages 4, 12).
	In study 4, the mean age of Iranian participants is 21.03, SD = 5.52, range 18-45 while for American participants it is = 40.01, SD = 13.42, range 19-79. This is a big difference and make the interpretation of the results questionable. These two samples comprise people from different generations, potentially with different values and priorities.	We thank Reviewer 1 for noting this important difference in samples' age. We added a discussion of the age difference in the Limitations section (page 30) and controlled for age in every study in our new analyses (pages 14, 18, 21, 25, 27).
	Study 1-3 study took place during the pandemic. study 4 data collection time in Iran was mid 2022 while it was early 2024 in the US. The results of self-reliance scores, therefore, might be influenced by the salience of disease and Covid health concerns. I suggest replicating the	We thank Reviewer 1 for this important comment. We agree that it is important to replicate this work in the post-COVID time as a robustness check. In this revision, we opted not to

	self-reliance findings in the post-covid time as a robustness check.	collect new data since it is not feasible for our research team to collect new data from Iran at the moment (there are no easy-to-use platforms like MTurk or Prolific in Iran available to researchers outside the country). However, we found differences in honor values in Studies 1-3 despite the ongoing pandemic and the salience of pandemic-related concerns. We think this suggests that, even when we hold pandemic concerns equal, there are still cross-cultural differences in the kinds of values that honor endorses. Nevertheless, we think that your point makes sense and we have added it to the Limitations section as something that needs to be addressed in future research (page 31).
	In study 4, researchers measured self-reliance moralization but not self-reliance itself. On the other hand, they measured family reliance but not family reliance moralization. What is the reason for this inconsistency? Wouldn't it be valuable to measure both types for both constructs?	We thank Reviewer 1 for this comment. In hindsight, there are certain measures that would have been useful to include, but we wanted to avoid administering too many measures, especially since the Iranian sample was not compensated for their time. Since we had tested honor-self-reliance associations previously, we opted to focus on self-reliance moralization. Part of our logic is that an honor-based endorsement of self-reliance values would be included within the moralization of self-reliance – honor would need to treat

		self-reliance as a moral concern before it can treat it as a moral good. We did not measure family-reliance moralization because we did not think of family-reliance as a personal ideal in the same way that self-reliance could be. We thought of family-reliance as something that was normalized by kinship values, but not something that people are told to strive toward. We have elaborated on family-reliance in the paper to make our conception of it more clear (page 22).
	The main argument of the paper is not clear in the introduction. My understanding is that self-reliance is argued to be the central theme in the version of honor prevalent in the US South. However, there are other equally important themes in the Iran's version. If this is the case, it needs to be clearly stated in the introduction.	Thank you for the suggestion. We agree and have revised the introduction to present our points more clearly (pages 3-4).
	The introduction begins with reputation and then transits rapidly to honor without establishing the link between the two: "Maintaining a good reputation is so vital that honor is arguably one of the three features of human culture [5], affecting how people engage with institutions [6,7] and other groups [8]. It's not clear why and how honor is related to reputation.	We agree and have removed mention of reputation from the introduction, as we now feel that it is tangential to our main points (pages 3-4).
	The term "moral conventions" and its relevance to the argument is not explained well. "This perspective allows self-reliance to be one of many means of demonstrating strength, with preference shaped by moral conventions [25,28]. Moral conventions specify the qualities that indicate prestige or superiority, thereby governing how honor relates to those qualities."	We apologize for the lack of clarity. We elaborate on this point further (page 5). We also changed "moral conventions" to "moral values" to make our wording consistent with other parts of the paper and avoid potential confusion.
	Study 2 and 3 are similar in their goals and methods. Therefore, I suggest rewriting them as study 2a and 2b.	We appreciate this suggestion. We have changed the study numbers. We believe this change has

		made our manuscript more fluent.
	On section 5.2.2. there is a mistake in reporting the correlation between honor scores and moralization of self-reliance: "Overall, honor scores correlated with endorsement of family-reliance, $r = 0.35$, $p < .001$, and moralization of self-reliance, $r = 0.12$, $p = .231$." The moralization of self-reliance correlation is not significant.	We apologize for this mistake. The section should now provide the correct numbers (page 25).
	The political orientation question (left-leaning vs right-leaning) does not seem to be diagnostics in the contemporary Iran for various reasons. Was this item pre-tested to ensure its validity?	Thank you for pointing this out. This item is very similar to what the World Value Survey (WVS) team has been using in Farsi. We did not pre-test this particular item, but we wanted a sense of how our participants chose to identify themselves politically. The fact that most of the participants considered themselves left-leaning was consistent with what we expected from Iranian participants recruited through social media.
	It would be beneficial to report the reliability (e.g. alphas) of the measures for each study in the main text.	Thank you for this suggestion. We have added the alphas in the methods section of each study.
Reviewer 2	1. Study 3 did not have attention checks, so no one was excluded from the analysis. Why did the authors decide to drop the attention check in this study? How did they ensure their data quality? How can they be certain that their participants paid enough attention in responding to the surveys, especially when data was recruited from social media, and participants did not receive any compensation?	Thank you for this comment. An attention check would have been useful; however, our reasoning was that, since participants had no external incentive to complete the study, they would be less likely to select arbitrary responses just to move ahead in the study.
	2. Table S2 supplementary materials: some measures across studies did not show what often considered in psychology good fit indices ($CFI > .90$, $TLI > .90$, $RMSEA < .06$-$.08$). I appreciate the fact that authors provided results on those measures that a two-factor or even three-factor	We thank Reviewer 2 for this comment. This is a good point, and we have included this in our limitations (page 31). We suspect part of the issue is that honor scales

	solutions showed better fits in supplementary, but in some cases, even two-three factor solutions showed relatively weak fits (e.g., Study 4 US. Honor Values, Study 4 Iran Self-Control Moralization). Interpretations, therefore, should be made with caution, and authors should point it out when discussing their limitations.	tend to draw on particular values, and there is likely heterogeneity in how people organize these values.
	3. The authors did a great job mentioning the potential limitations of applying this study's findings to other racial-ethnic minorities in the US (e.g., African Americans). While they rightly acknowledge the United States' racial and ethnic diversity, they do not discuss Iran's ethnic diversity. Iran's ethnic composition includes various ethnic groups, such as Persians, Azeris (Turkish), Kurds, Lurs, etc. Therefore, ignoring this diversity and generalizing findings from Persian Iranians (as reported in Table 1, 100% of the sample was Persian and Farsi was their first language) to all Iranians is not a good research practice and should be noticed. We should never assume the other, non-American context is homogeneous, thus avoiding the out-group homogeneity bias.	We are glad that the reviewer thinks we have done a great job mentioning the limitations of our cross-cultural research. We revised the section to include heterogeneity in Iran as well, with Kurds as an example of a minority group whose cultural history did not follow the same trajectory as the Persian majority (pages 30-31).
	4. My previous experiences with collecting data from Iranian social media have shown that the sample you usually get is highly educated, liberal (as in study 3), young, has a high rate of people from average to high SES, and is biased toward females (as in study 3 & 4). Therefore, the sample might be different from the actual Iranian demographic. A representative sample may bolster this study's findings, as samples from Iranian social media users tend to be similar to WEIRD populations. Still, it would be highly appreciated if the authors could also mention this as a limitation.	We thank the reviewer for bringing this point up. We fully agree. We have added this as a limitation (page 30).
	5. Age in Study 4: As shown in Table 1, the mean age difference between the American and Iranian samples is ~19 years old, which is not controlled for in Study 4! It is also possible that differences exist in the political orientations of the two samples (related to my comment above on problems with samples from Iranian social media). As shown in Table 1, the mean political orientation of the American sample is M = 3.23.	These are very helpful suggestions. We have added age as well as other available demographic variables as controls in all of our analyses (pages 14, 18, 21, 25, 27). These variables generally did not change our pattern of results, but we also decided

	Although the authors did not report the same construct for the Iranian sample (my first comment is minor concerns), based on the information provided in study 3, we would expect a highly liberal sample of Iranians. I'm unsure if the authors collected other demographics (it seems they have collected education levels from Americans and not Iranians, and it is not clear why). Still, it is highly advised to control for all the possible demographics when comparing samples from two cultures and to report them either in the main manuscript or supplementary materials.	to mention age differences as a concern in limitations (page 30). We collected education levels from Iranians in the final study because one of our authors wanted to include it for exploratory purposes. We did not intend to use it for this project, but we decided to control for it as well for the Iranian sample.
	1. Table 1: Information about the political orientation of the Iranian sample needs to be included in Study 4.	Thank you for this comment. Due to researcher error, we failed to include a political orientation item for Iranians in that study. It is likely that we failed to transfer the item over when we were preparing a new set of measures. It is likely that the new sample was also predominantly left-leaning, but we cannot say for sure.
	2. Instead of presenting the same mediation model three times in three different figures, the authors could provide one figure in which the conceptual mediation model is explained and then report the findings in the manuscript (because they will report them in the manuscript anyway). This would save a lot of space.	We thank Reviewer 2 for this important and helpful comment. We have incorporated this by setting up one conceptual mediation model figure (page 15) and referring to it by path labels in the results (pages 14-15, 18, 21).
	3. The same could also be applied to regression tables from study 1 to study 3 (tables 3, 5, and 6).	We thank Reviewer 2 for this suggestion. We have merged the three tables into one (page 13).
